# Microclimatic regulation effects of landscape elements on university campus in a temperate monsoon climate zone: A case study during extreme summer heat

**DOI:** 10.1371/journal.pone.0362099

**Published:** 2026-11-05

**Authors:** Lei Fan, Yixin Liang, Zixian Li, Yan Zhou

**Affiliations:** 1 Forestry College, Shenyang Agricultural University, Shenyang, China; 2 Key Laboratory of Northern Landscape Plants and Regional Landscape, Shenyang, China; Changan University: Chang’an University, CHINA

## Abstract

Although temperate monsoon climate regions are traditionally known for their harsh winter climates, the combined effects of rapid urbanization and global climate change have significantly intensified summer urban heat island effects in these areas, making them a neglected yet increasingly severe challenge in high-latitude urban climate adaptation planning. This study takes a typical campus in Shenyang, a temperate monsoon climate region in Northeast China, as the experimental subject. It quantitatively dissects the non-linear statistical associations and regulatory mechanisms of biotic and abiotic landscape elements and their spatial configurations on microclimate parameters and human thermal comfort (Physiologically Equivalent Temperature, PET) by combining microclimate field observations with validated ENVI-met numerical simulations. Field measurement results show that the vertical structural characteristics of vegetation dominate cooling efficiency: evergreen trees and shrubs, with their dense radiation-blocking structures, exhibit optimal regulatory potential, with mean Physiological Equivalent Temperature (ΔPET) values reaching 10.6°C and 10.4°C, respectively, significantly outperforming deciduous trees (8.1°C). In contrast, abiotic elements play an auxiliary role, with dynamic water bodies and enclosed pavilions providing PET improvement values of 3.1°C and 2.9°C, respectively, while paving materials have limited direct regulatory capacity (< 2.0°C). Simulation optimization further reveals a significant “blue-green synergy” effect: the composite configuration of high-canopy evergreen trees and large-area dynamic water bodies forms the optimal spatial solution, reducing daily average PET to 48.7°C, a decrease of 7.3°C compared to the vegetation-free baseline scenario. Addressing the unique “humid-heat” challenge that distinguishes temperate monsoon climates from arid or semi-arid regions where summer thermal stress is often attributed solely to high temperatures, this study goes beyond merely documenting cooling effects to elucidate the “structural-thermal decoupling” mechanism in temperate monsoon climates. It challenges the conventional “deciduous-first” design paradigm by quantifying the superior radiative-blocking efficiency of evergreen species during extreme heatwaves, providing a physically-grounded framework for campus microclimate resilience.

## 1. Introduction

Global warming precipitates not only environmental degradation but also profound adverse effects on human health. The prevalence of associated neurodegenerative diseases is projected to rise concomitantly with increasing global temperatures [1]. Multiple studies indicate that elevated temperatures exacerbate both all-cause and cardiovascular mortality rates [2]. For instance, the European heatwave of summer 2003 was estimated to have caused approximately 40,000 excess deaths [3]. In numerous regions, heat-related mortality events previously categorized as centennial occurrences under the climatic conditions of the 2000s are projected to recur every 10–20 years. These recurrence intervals are anticipated to contract further as global warming reaches thresholds of 1.5°C and 2.0°C. Absent effective adaptation strategies, mortality events historically driven by extreme heat are poised to become the norm [4]. Furthermore, unchecked global warming threatens a precipitous decline in food production, potentially resulting in up to 6 billion fatalities by 2100 [5]. Moreover, as China’s rate of surface warming exceeds the global average, the frequency of extreme heat events is projected to escalate. Accounting for improvements in adaptive capacity, the annual heat-related mortality rate—recorded at 32.1 per million during 1986–2005—is projected to rise to 48.8–67.1 per million at 1.5°C warming, and 59.2–81.3 per million at 2.0°C warming [6].

The Urban Heat Island (UHI) phenomenon is intensified by rapid urbanization and economic development, processes which are fundamentally driven by burgeoning urban populations [7]. Consequently, elevated urban temperatures render inhabitants of cities and surrounding areas significantly more susceptible to heat-related illnesses [8]. To address this, extensive research has been conducted at both urban and micro-scale levels to enhance human thermal comfort. Investigations have focused on the efficacy of high-albedo paving materials [9,10], the impact of shaded spaces [11], and the potential of vegetation [12–19]. Among these strategies, the deployment of vegetation is widely regarded as paramount for mitigating heat stress. Findings indicate that trees contribute to cooling through two primary mechanisms: shading and evapotranspiration. First, the canopy lowers temperatures in the sub-canopy and adjacent areas by absorbing, reflecting, and transmitting shortwave radiation [20–23]. Second, through transpiration, stomata release water vapor, thereby dissipating heat and cooling the ambient environment [24–28]. This cooling efficacy is observed at the level of both individual specimens [12] and aggregate tree clusters [29,30]. However, impact varies by species. Statistical analyses utilizing the Sky View Factor (SVF)—derived via hemispherical photography—demonstrate that SVF significantly influences tree surface temperatures. Furthermore, studies characterizing tree morphology through Leaf Area Index (LAI) reveal a positive correlation between LAI and cooling intensity: higher LAI values correspond to more pronounced cooling effects [26,30,31].

However, research regarding summer tree cooling within China’s Temperate Monsoon Climate Region remains relatively underexplored. The scientific rationale for investigating summer heat stress in a temperate monsoon climate zone is rooted in the region’s distinct “humid-heat” characteristics. Unlike arid or semi-arid regions where high temperatures are the primary driver of thermal discomfort, temperate monsoon climates experience concurrent high temperature and high relative humidity during summer due to moisture advection from adjacent seas via the East Asian summer monsoon. This humidity exacerbates heat stress by suppressing evaporative cooling from the human body, as the air’s moisture-holding capacity limits sweat evaporation—a mechanism fundamentally different from “dry heat” stress. As documented by Clark et al., excessive relative humidity in combination with high temperature can lead to dangerous levels of the Wet-Bulb Globe Temperature (WBGT), even at moderate absolute temperatures. Furthermore, under global warming, modeling projections indicate that eastern Asia, including China’s temperate monsoon regions, will experience increasingly severe humid-heat extremes, with WBGT increases of 5–7°C by the end of the 21st century under a high emissions scenario. Therefore, studying landscape strategies to mitigate summer thermal discomfort in temperate monsoon climates—where both radiative (solar) and convective (humidity) stresses coexist—is not merely an extension of heat mitigation research from warmer climates, but addresses a distinct biometeorological challenge with unique physical mechanisms (i.e., the coupling of shortwave radiation blocking and humidity-sensitive thermal comfort evaluation). Diverging from the linear paradigm of southern regions—which prioritizes year-round shading—the Temperate Monsoon Climate Region confronts a unique “seasonal trade-off”: the imperative to maximize solar gain during winter while simultaneously mitigating heat stress induced by short-term high temperatures and intense radiation in summer. Addressing this complexity, several studies over the past five years have provided critical empirical evidence. First, concerning plant functional types, the comparative efficacy of evergreen versus deciduous trees has emerged as a central focus. Utilizing a combination of in situ measurements and ENVI-met simulations at Shenyang Agricultural University, findings indicate that during periods of peak summer solar radiation, evergreen stands reduce air temperatures by 2.1–3.4°C and increase relative humidity by 8–12%RH compared to open areas. This significantly outperforms deciduous trees, which yielded an average reduction of only 1.2°C under identical conditions. This performance advantage is primarily attributed to the persistent canopy structure of evergreens, characterized by a higher Leaf Area Index (LAI) and lower Sky View Factor (SVF), which enhance both shading efficiency and transpirational cooling [32]. Further corroborating these findings, research conducted in Harbin demonstrated that blue-green spaces in temperate monsoon climate zones generate a distinct “Cold Island Effect” during summer; temperatures within a 6–8 meter radius of water bodies decreased by 1.8–2.5°C, with thermal regulation effects amplified when integrated with vegetation [33].

Secondly, regarding the coupling mechanisms between individual and composite elements, recent scholarship has elucidated the synergistic effects inherent in landscape configurations. An ENVI-met simulation focusing on campus courtyards demonstrates that when arborous coverage exceeds 30% and is paired with high-albedo permeable paving, the Physiological Equivalent Temperature (PET) can be reduced by over 9.5°C compared to a baseline scenario of asphalt paving without shade [34]. Furthermore, the spatial integration of blue and green infrastructure exhibits significant synergistic cooling capacity; studies indicate that positioning trees along water margins can augment the magnitude of PET reduction in surrounding areas by an additional 15–20% (proportional increase) [35]. While existing frameworks often assume a linear correlation between green volume and cooling, this research targets the non-linear thresholds where urban morphology and vegetation transpiration interact. We specifically investigate the “synergistic cooling increment” of blue-green spatial coupling, a critical yet under-explored dimension in high-density campus environments.

However, extant literature has predominantly concentrated on the cooling effects of isolated vegetation types, lacking a systematic deconstruction of the comprehensive regulatory chain encompassing “efficacy grading of individual elements—mechanisms driven by morphological parameters—and synergistic enhancement of composite spaces.” This deficiency is particularly pronounced within the specific climatic context of the Temperate Monsoon Climate Region, characterized by high heat and humidity, where achieving optimal thermal comfort through the precise configuration of landscape elements remains an unresolved challenge. To bridge this gap, this study employs a hybrid approach combining in situ measurements and numerical simulations to establish an adaptive regulation framework that spans from micro-scale mechanisms to macro-scale spatial layouts. Specifically, it addresses three progressive critical scientific questions:

(1) Quantification: Under typical conditions of high heat and humidity, what are the regulatory thresholds and hierarchical distinctions among various biotic (trees, shrubs, grass) and abiotic (water bodies, paving) landscape elements regarding microclimate and human thermal comfort (PET)?
(2) Mechanism: How do canopy structural parameters (e.g., LAI, SVF) drive non-linear variations in microclimatic factors, and what are the underlying physical mechanisms governing these interactions?
(3) Optimization: Within spatially constrained campus environments, how can the “blue-green synergistic” configuration of vegetative and abiotic elements be leveraged to maximize additive cooling effects, thereby providing quantitative strategies for climate-adaptive design in the Temperate Monsoon Climate Region?

## 2. Materials and methods

### 2.1. Overview of the study area

This study is sited in Shenyang (41°11′–43°20′N, 122°25′–124°18′E), a quintessential representative city within the Temperate Monsoon Climate Region of Northeast China. Characterized by a temperate continental monsoon climate with distinct seasonal variations, the region—despite its protracted and frigid winters—experiences high temperatures and high relative humidity (RH) during summer due to monsoon influences. Average maximum temperatures in July frequently exceed 30°C, with RH often exceeding 80% during the day and approaching saturation at night. This combination of high temperature and elevated humidity creates a “humid-heat” environment that compromises human thermoregulation by reducing evaporative cooling efficiency—a phenomenon distinct from the “dry heat” stress typical of Mediterranean or semi-arid climates. Consequently, outdoor thermal discomfort in Shenyang during summer is driven not only by solar radiation (as measured by PET) but also by humidity-sensitive heat stress, rendering it an increasingly salient issue under global warming [36]. Recent urban heat island research in Shenyang has documented significant nighttime heat stress exacerbated by high humidity, with maximum urban heat island intensities reaching 7.6°C in built-up Local Climate Zones. Field observations were conducted at “Kewei Garden,” a core landscape zone within the campus of Shenyang Agricultural University. Spanning an area of approximately 4.9 hectares, the site extends roughly 190 m from east to west and 260 m from north to south. The study was conducted within the public campus areas of Shenyang Agricultural University. Field site access was granted via oral permission from the Logistics Management Department of Shenyang Agricultural University. No specific permits were required as the study did not involve private land, protected areas, or endangered/protected species. The site features a rich diversity of typical landscape elements, comprising complex multi-layered plant communities (arbor-shrub-herb structures), heterogeneous impervious surfaces (asphalt, granite, Dutch brick, etc.), varied water features, and artificial shading structures. Consequently, it serves as a representative model of outdoor spaces in universities within temperate monsoon climate zones, offering an ideal experimental testbed for investigating microclimatic interaction mechanisms [37–39].

### 2.2. On-site field test experiment design

#### 2.2.1. Typical measurement point selection.

To systematically quantify the microclimatic regulatory effects of diverse landscape elements, this study established a monitoring network based on a hierarchical logic progressing from individual elements to composite communities. Regarding the selection of Plant Functional Types (PFTs), we chose 14 representative species common to Shenyang. These encompassed five evergreen tree species, seven deciduous tree species, and two shrub species. Selection criteria required that each specimen be mature and free of pests or diseases. To quantify cooling magnitude, paired measurement points were established at the geometric center of the canopy shade and at an adjacent unshaded reference location [40,41]. Detailed tree specifications and schematic diagrams of measurement positions are provided in S1 Fig, and S1 Table. Concurrently, categorical monitoring points were designated for abiotic elements. These included underlying surfaces with varying albedo values such as dark asphalt and light granite, diverse water body morphologies including dynamic fountains and static lakes, and artificial shading structures like enclosed pavilions and permeable pergolas. Environmental characteristics and hemispherical photographic records of the landscape element monitoring points are detailed in S2 Table. Furthermore, to investigate synergistic interactions among elements, specific composite habitat monitoring points were established. These configurations included trees paired with water bodies, trees integrated with paving, and trees combined with shading structures. All monitoring points were situated in typical open areas devoid of anthropogenic heat interference to ensure data independence and representativeness [10,42,43].

#### 2.2.2. Meteorological data collection and instrument configuration.

Field campaigns were conducted on a representative summer day (July 11, 2024) characterized by clear skies and low wind speeds, with meteorological conditions closely matching the July climatological normals for Shenyang. The monitoring window spanned from 07:00–19:00 to capture the most significant diurnal fluctuations in the thermal environment. Data acquisition for microclimatic parameters was standardized at a height of 1.5 m above ground level. This elevation was selected to approximate the center of gravity of a standing human and the core zone of thermal perception [44,45]. A suite of precision portable instruments was employed for data collection. Air temperature, relative humidity, and wind speed were recorded using a Kestrel 4500 Pocket Weather Tracker (Nielsen-Kellerman, USA), which boasts accuracies of ±1.0°C, ± 3% RH, and ±3% of reading respectively [46]. Globe temperature, serving as a proxy for radiant heat load, was measured via a WBGT-213BN Heat Index Meter. Synchronous monitoring of solar shortwave radiation intensity was achieved using a TES-1333R Solar Power Meter, while mean surface temperatures were determined using an H21Pro infrared thermal imager with an accuracy of ±2°C. Additionally, hemispherical imagery was captured using a Canon EOS 6D camera equipped with a 180° fisheye lens to facilitate the subsequent calculation of the Sky View Factor (SVF). Canopy structural parameters, specifically the Leaf Area Index (LAI), were acquired using an LAI-2200C Plant Canopy Analyzer (LI-COR, USA). These measurements were taken after sunset using a multi-point averaging method to ensure data reliability [47]. Prior to deployment, all instruments underwent unified cross-calibration to eliminate potential systematic errors.

To justify the representativeness of the selected observation day, we compared the meteorological conditions on July 11, 2024, with the July climatological normals for Shenyang. According to historical data from the China Meteorological Administration, the July average daily maximum temperature in Shenyang ranges from approximately 29°C to 31°C, while the average daily minimum ranges from 19°C to 21°C. The observed values on July 11, 2024 (maximum 31.9°C, minimum 20.13°C), fall within this typical range. Furthermore, July 11 featured a typical summer synoptic pattern dominated by a subtropical high-pressure system, resulting in clear skies and low wind speeds (< 2.0 m/s) — conditions that maximize solar radiation and heat stress, making it an ideal case for assessing the upper bound of landscape cooling efficacy. While single-day observation cannot capture full seasonal variability, this day represents a worst-case scenario for summer thermal discomfort, providing a conservative benchmark for evaluating landscape mitigation strategies.

#### 2.2.3. Thermal comfort assessment model.

Physiological Equivalent Temperature (PET) was employed as the definitive metric for assessing outdoor thermal comfort, as it synthesizes the combined influence of air temperature, humidity, wind velocity, and radiation on human thermal balance [45]. Computational procedures were facilitated by the RayMan Pro model, incorporating input parameters such as measured air temperature, relative humidity, wind velocity, and mean radiant temperature (Tmrt). Specifically, the mean radiant temperature was derived in accordance with the ISO 7726 standard, calculated from measured globe temperature in conjunction with air temperature and wind velocity [44]. To maintain consistency in evaluation baselines, personal parameters within the model were standardized to a hypothetical 35-year-old male with a height of 1.75 m and a weight of 75 kg. This standard subject was characterized as wearing light attire typical of summer, with a thermal resistance of 0.5 clo, and engaged in light physical activity corresponding to a metabolic rate of 80 W [45,48]. To rigorously examine the causal linkage between radiative flux and thermal comfort, a sensitivity analysis was conducted. By isolating the Mean Radiant Temperature (Tmrt) from other convective parameters (e.g., wind speed and relative humidity) within the RayMan model, we quantified the marginal contribution of net radiation to PET fluctuations, ensuring that the identified ‘dominance’ is physically grounded rather than merely statistical.

### 2.3. Numerical simulation methods and model validation

#### 2.3.1. Model construction and parameter settings.

Kewei Garden was selected as the representative simulation domain, where a three-dimensional microclimate model was constructed utilizing data derived from field surveys [49]. The spatial dimensions of the model domain were established at 190 m x 260 m x 70 m. To achieve an optimal equilibrium between computational precision and efficiency, a grid resolution of 2 m x 2 m x 2 m was adopted [50]. Underlying surface materials were mapped to specific physical categories such as loam, asphalt, red brick, yellow brick, water bodies, granite, and concrete in accordance with on-site attributes [43]. Regarding vegetation modeling, 44 recorded species—including elm, fir, and lilac—underwent digital reconstruction based on measured morphological parameters such as tree height, crown width, and Leaf Area Density (LAD). For species not indexed within the software database, a “morphological-functional substitution” method was employed. This approach involved selecting analogous species from the 3D plant library (specifically Abstract Deciduous or Conifers) that possessed similar heights and consistent canopy geometries. This parameterization strategy was critical to ensure the fidelity of canopy radiative transfer and aerodynamic simulations [51,52].

#### 2.3.2. Boundary conditions and simulation runs.

The initial meteorological forcing files were generated relying on in situ measurements derived from the campus weather station, encompassing critical parameters including hourly air temperature, relative humidity, wind speed, and wind direction. To capture the complete diurnal cycle of summer solar radiation, the simulation duration was established as a 12-hour window extending from 07:00–19:00. Analysis of the initial background meteorological conditions indicated that the selected simulation date of July 11, 2024, exemplified weather typical of high-temperature and high-humidity climates. Specifically, the maximum air temperature reached 31.9°C while the minimum recorded was 20.13°C, with relative humidity fluctuating between 55.05% and 100%.

The initial and boundary conditions for the ENVI-met simulations were defined based on on-site measurements from the campus weather station on July 11, 2024, which represented typical summer conditions characterized by high temperature, high humidity, and clear skies. The simulation domain was configured with a 12-hour period from 07:00–19:00 local time to capture the full diurnal cycle of solar radiation and thermal dynamics.

Initial conditions were specified at the start of the simulation (07:00) based on observed data, including air temperature, relative humidity, wind speed and direction, and soil temperature and moisture profiles. Boundary conditions were applied to the lateral and top boundaries of the model domain to drive the simulation over time. The meteorological forcing file was generated using hourly input data derived from the in-situ measurements. Table 1 summarizes all key initial and boundary condition parameters used in the simulations.

**Table 1:** Summarizes all key initial and boundary condition parameters used in the simulations.

Parameter Category	Parameter	Value
**Simulation domain**	Model size (grid cells)	190 (x) × 260 (y) × 70 (z)
	Grid resolution	dx = dy = 2 m, dz = 2 m
	Nesting grids	5
	Simulation period	08:00–19:00, July 11, 2023
**Initial meteorological conditions (at 07:00)**	Air temperature (Ta)	23.163°C
	Relative humidity (RH)	89.80%
	Wind speed (Va)	0.3 m/s
	Wind direction	45° (South)
	Specific humidity at 2m	0.0158 kg/kg
**Boundary conditions**	Lateral boundary condition	Open (cyclic)
	Top boundary condition	Forced (K-type)
	Roughness length (z0)	0.1 m
**Soil initial conditions**	Soil type	Loam (0–50 cm)
	Initial soil temperature (upper layer)	20.0°C
	Initial soil moisture (upper layer)	0.15 (m³/m³)
**Radiative conditions**	Solar adjustment factor	1
	Longwave radiation adjustment	0.9
	Albedo of surrounding surfaces	0.20 (average)

#### 2.3.3. Model validation.

To verify the applicability of the ENVI-met model for summer conditions in the temperate monsoon climate zones of Shenyang, the Root Mean Square Error (RMSE) was adopted as the quantitative validation metric [53]. Three representative monitoring sites within Kewei Garden were selected to conduct a fitting analysis between simulated outputs and measured data. This analysis covered the time interval extending from 13:00–18:00.

Validation results indicated that the RMSE for air temperature was 2.27°C, while the RMSE for relative humidity stood at 8.96% RH [53]. According to universal standards in the field of microclimate simulation, which regard an air temperature RMSE ranging from 0.52°C to 4.30°C and a humidity RMSE varying between 2.04% RH and 10.2% RH as acceptable, the error metrics of this model fell entirely within the valid range. Although minor deviations arose from handheld instrument measurement errors (specifically ±1°C for temperature and ±3% RH for humidity) and spatial heterogeneity between the weather station and the site, the overall trends exhibited high consistency. This confirms that the model is capable of accurately reproducing the microclimatic thermal processes of the study area, thereby validating its utility for subsequent spatial optimization simulations [53–55].

### 2.4. Data statistical analysis

All empirical microclimate data and simulation outputs underwent rigorous organization and cleansing processes, culminating in the construction of a foundational database via Microsoft Excel. Statistical analyses regarding significance were conducted utilizing SPSS 26.0 software (IBM Corp., USA). Specifically, One-way Analysis of Variance (One-way ANOVA) was employed to evaluate cooling disparities among distinct Plant Functional Types and landscape elements. To elucidate the coupling mechanisms inherent in the “structure-process-function” paradigm, Pearson correlation analysis was applied to quantify the strength of linear associations between canopy structural parameters (such as LAI and SVF) and both microclimatic factors (including Ta and T_mrt_) and thermal comfort indices (PET) [56,57]. For all statistical tests, the threshold for significance was established at P < 0.05 [58,59].

## 3. Results

### 3.1. Hierarchy of microclimate regulation efficiency of landscape elements

#### 3.1.1 Regulatory differences in plant functional types: Absolute dominance of trees and near-ground effects of shrubs.

Overall, Evergreen Trees exhibited the optimal thermal amelioration efficacy, attributable to their high-density needle canopy structures. During the midday period extending from 12:00–14:00, the reduction in Physiological Equivalent Temperature (ΔPET) beneath their shade reached a substantial average of 10.6°C. Notably, the Shrub community demonstrated a counter-intuitive “shrub surpassing” phenomenon; the mean empirical ΔPET of 10.4°C for shrubs was statistically superior to that of the Deciduous Trees group, which recorded 8.1°C. This finding suggests that within the height range of human core thermal perception (1.5 m), shrubs construct an efficient near-surface radiation barrier through dense foliage proximate to the ground. This structure effectively intercepts terrestrial long-wave radiation and suppresses near-surface sensible heat flux. Conversely, certain deciduous species were constrained by elevated branching points and sparse canopies, which resulted in higher optical transmissivity and consequently limited thermal regulation potential.

While various plant categories generally adhere to the aforementioned patterns, results from multi-dimensional heat maps also unveil significant interspecific heterogeneity in parameters and functional trade-offs (Fig 1). To be specific, although Evergreen Trees generally yielded the optimal improvement effects, certain species such as *Juniperus chinensis* exhibited elevated canopy surface temperatures reaching 29.1°C due to leaf morphology. Consequently, the Physiological Equivalent Temperature in sun-exposed areas (PET_sun_) for this species peaked at 49.9°C, the highest within the entire sample. This indicates that while evergreen species provide efficient shading, their heated surfaces may simultaneously function as localized secondary heat sources. In contrast, *Salix babylonica* and *Populus tomentosa* within the Deciduous Trees group adopted a more balanced strategy. These species not only maintained lower canopy temperatures fluctuating between 28.5°C and 28.6°C but also significantly controlled PET_sun_ within a range extending from 39.5°C to 40.3°C, thereby demonstrating superior comprehensive capability for microclimate improvement. Furthermore, while the shrub layer excelled in radiant interception as previously noted, its physiological cooling capacity exhibited distinct limitations. Citing *Syringa oblata* as an instance, even within a relatively mild environmental context, its leaf-to-air temperature difference (ΔT_lc-a_) was merely −2.8°C. This value is considerably inferior to that of the arbor layer, suggesting that low-stature shrubs face difficulties in achieving efficient active cooling solely through endogenous transpiration when devoid of shading from an upper tree canopy.

**Fig 1:**
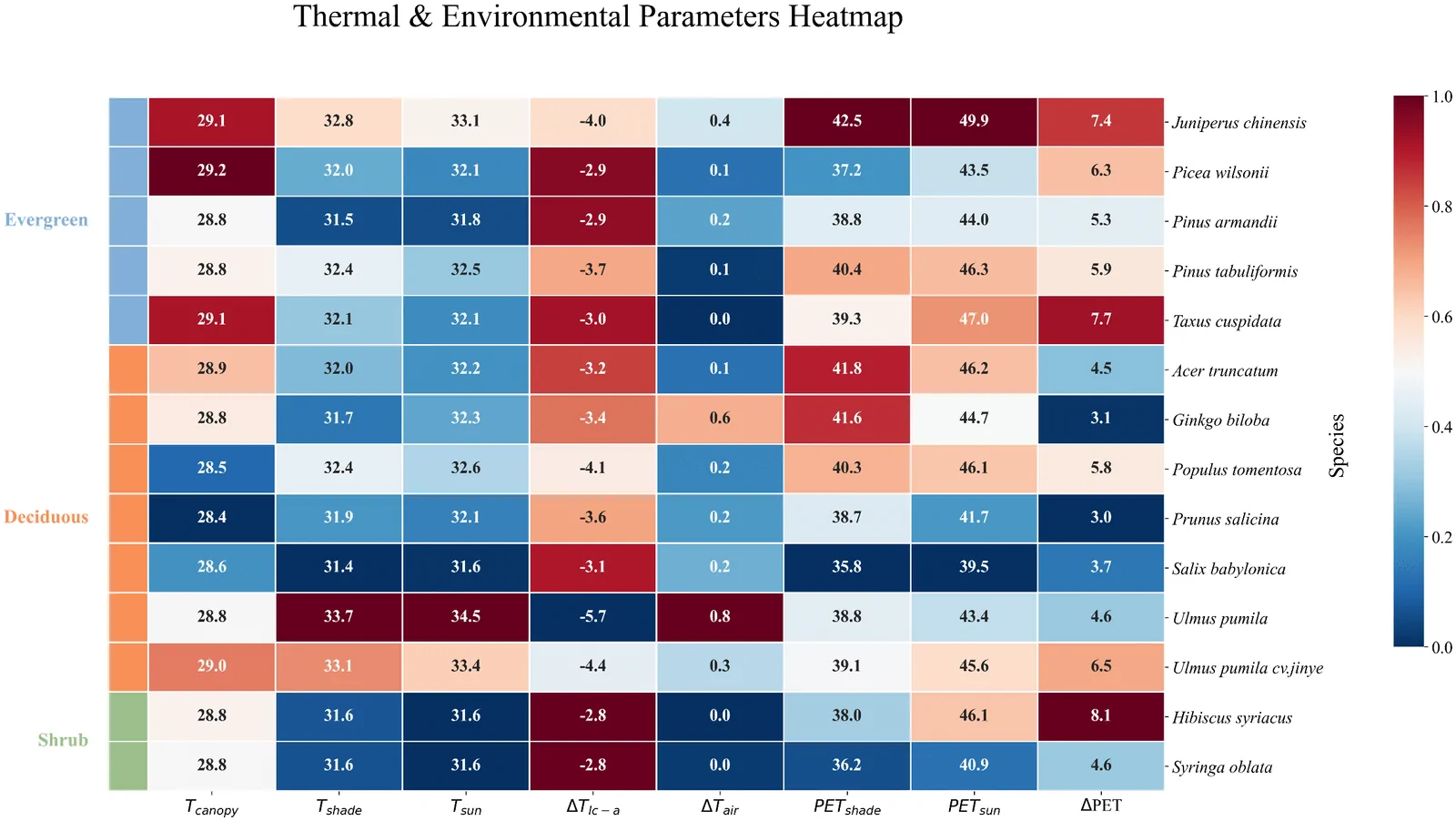
Heatmap of multidimensional thermal physiological parameters and microclimatic factors for 14 plant species. Cell colors represent the relative rank of each metric across all species (based on 0–1 min-max normalization, where red indicates high values and blue indicates low values). Numbers within cells denote the actual observed mean values (Units: °C for temperature and PET).

Fig 2 quantitatively compares the impacts of 11 distinct landscape element configurations on the improvement of ΔPET, thereby establishing the absolute dominance of natural vegetation in thermal environmental regulation. Overall, solitary vegetation communities exhibited cooling efficacies significantly superior to those of artificial facilities and compound landscapes. Specifically, Evergreen Trees and Shrubs demonstrated the highest regulatory potential, with their mean ΔPET values reaching 6.5°C and 6.3°C, respectively. This magnitude is approximately 1.5 times that of Deciduous Trees, which recorded a value of approximately 4.4°C. Notably, compound landscape elements (such as Tree + Water and Tree + Shade Device) did not exhibit the expected “additive cooling effect.” Their magnitudes of cooling were generally inferior to those of solitary high-density Evergreen Trees. This phenomenon may be attributed to restricted canopy specifications or insufficient spatial closure within compound scenarios. This suggests that within limited spaces, the simple accumulation of landscape elements does not necessarily surpass the radiative blocking benefits yielded by high-density vegetation.

**Fig 2:**
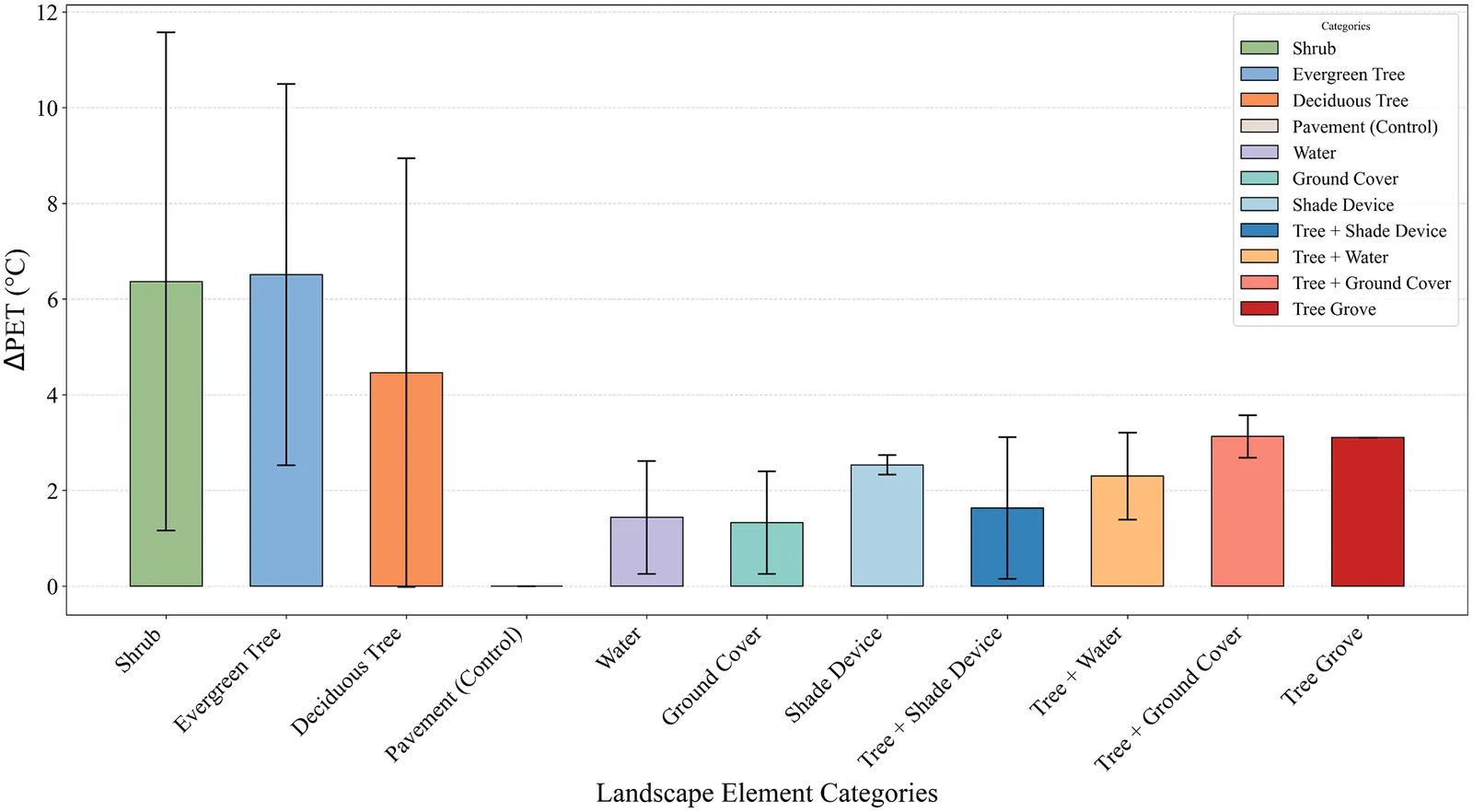
Quantification of microclimatic regulation performance (ΔPET) for single and combined landscape elements.

#### 3.1.2. Auxiliary regulation of abiotic elements: Thermal correction of water body morphology and paving materials.

Supplementing the active regulation provided by plant communities, abiotic underlying surfaces distributed extensively across the campus constitute the physical substrate of the microclimatic environment, where their thermal performance exerts a significant modifying influence on local thermal conditions (Table 2). Variations in material albedo and heat capacity characteristics drive distinctly divergent processes of surface energy balance. Empirical data indicate that dark asphalt pavements characterized by low albedo exhibit a potent “sensible heat source” effect during midday periods of high radiation. By continuously transferring heat to the human body in the form of long-wave radiation, these surfaces result in PET values significantly exceeding those of natural grasslands, thereby establishing zones of heat stress. In contrast, high-albedo granite and permeable brick pavements, while lacking the evaporative cooling mechanisms of vegetation, effectively suppress surface temperature elevation through their high reflectivity regarding solar short-wave radiation. Within open hardscape plazas devoid of arborous shading, this “Cool Pavement” strategy serves as a pivotal passive regulatory measure for mitigating heat stress by reducing the long-wave radiant load originating from the ground surface.

**Table 2:** Statistics of daily mean Physiological Equivalent Temperature (PET) and improvement magnitude (ΔPET) for different landscape element measurement points).

	Measurement Point	Daily Mean PET (°C)	ΔPET (PET _pavement_-PET _measurement point_)
**Pavement (as a control group)**	a0	37.6	0
	b0	36.6	0
	c0	36.1	0
	d0	38.7	0
	e0	39.5	0
	f0	37.9	0
**Water body**	d2	39.4	0.7
	e1	36.4	3.1
	f2	37.4	0.5
**Groundcover plants**	a1	37.9	−0.3
	a2	37.9	−0.3
	b1	34.7	1.9
	b2	33.8	2.8
**Shade device**	c1	33.3	2.8
	d1	36.2	2.5
	f1	35.6	2.3
**Trees and shading devices**	C1	35.8	0.3
	D1	37.8	0.9
	F1	34.2	3.7
**Trees and water bodies**	A1	35.1	2.5
	D2	37.6	1.1
	E1	36.2	3.3
**Trees and groundcover plants**	A2	35	2.6
	A3	34.7	2.9
	B1	33.4	3.2
	B2	32.8	3.8
**Thicket space**	D3	35.6	3.1

In terms of microclimate modification within vertical space, water bodies and artificial structures exert cooling effects through two distinct pathways: “enhanced latent heat flux” and “mechanical radiative interception,” respectively. Furthermore, their efficiency is highly dependent on morphological characteristics. Although the overall regulatory capacity of abiotic elements is limited (with mean values maintaining a range from 1.3°C to 2.5°C), optimization of specific morphologies can significantly elevate their performance. For instance, dynamic water features such as large fountains substantially increase the water-air contact interface through droplet fragmentation. This process intensifies the efficiency of heat absorption during phase changes inherent in evaporative cooling. Consequently, the ΔPET for dynamic water reached 3.1°C, a value significantly superior to that of static water surfaces which rely solely on surface evaporation. Regarding artificial shading facilities, enclosed pavilions with solid roofing provided a ΔPET improvement of 2.9°C, outperforming pergolas covered by vines with permeable tops. While pergolas offer a degree of shelter, their relatively high Sky View Factor (SVF) still permits the penetration of partial direct radiation; in contrast, pavilions completely intercept the input of solar short-wave radiation. This result resonates with the high efficacy observed in Evergreen Trees, reaffirming from the perspective of abiotic elements that “Radiative Blocking” constitutes the primary dominant mechanism for improving outdoor thermal comfort in summer.

### 3.2. Non-linear driving mechanism of canopy structure on thermal comfort

#### 3.2.1. Decoupling phenomenon of canopy thermal characteristics: Surface temperature (T_canopy_) is not the main cause of cooling.

Collectively, all plants subjected to measurement exhibited a distinct Canopy Cooling Effect. As illustrated in Fig 3, under conditions characterized by a relatively uniform local microclimate (where T_sun_ and T_shade_ were maintained within a range extending from 31.56°C to 32.67°C), the canopy surface temperature (T_canopy_) descended significantly to a level varying between 28.74°C and 29.02°C. This cooling capacity was particularly pronounced within the arbor layer. Despite the fact that Evergreen and Deciduous Trees were subjected to environmental heat loads slightly exceeding those experienced by shrubs, they generated a maximum temperature differential reaching as high as 3.93°C (specifically in Deciduous Trees). This finding implies that the arbor layer possesses a more robust thermal regulatory potential than that of the shrub layer, which exhibited a differential of approximately 2.77°C.

**Fig 3:**
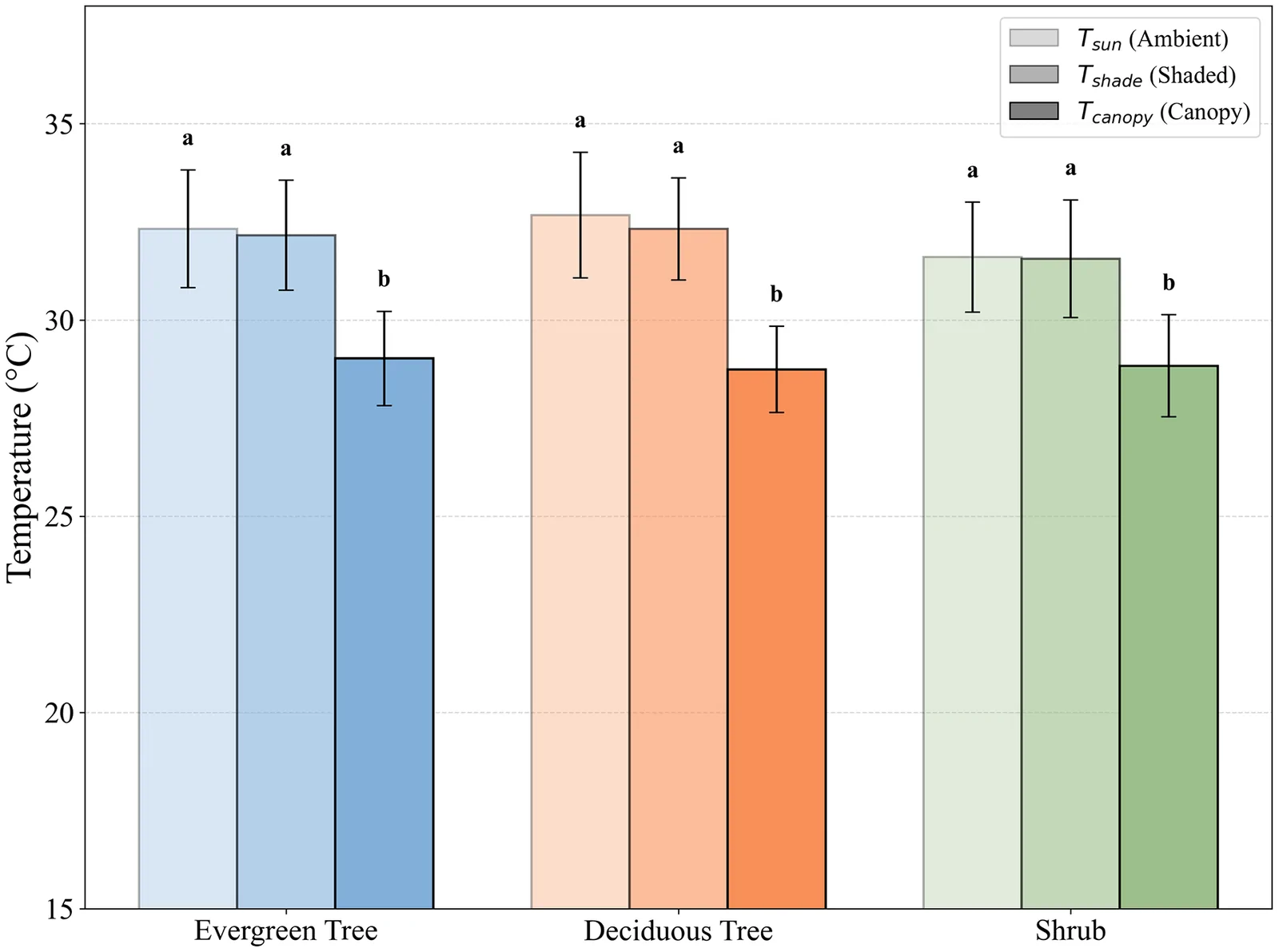
Comparison of canopy surface temperature (T_canopy_) and ambient air temperatures (T_sun_ and T_shade_) across three plant functional types.

However, a comprehensive interspecific analysis, as depicted in the box-plots of Fig 4, unveiled significant heterogeneity in thermal responses. Evergreen Trees typically exhibited a relatively narrow distributional range for canopy temperature (T_canopy_), which indicates a comparative stability within their canopy thermal environments. In contrast, certain species within the Deciduous Tree and Shrub categories, such as *Salix babylonica* and *Syringa oblata*, demonstrated larger Interquartile Ranges (IQR). This suggests a heightened variability in the responses of these species to dynamic environmental thermal conditions. To be specific, *Juniperus chinensis* displayed a higher median temperature among Evergreen Trees, whereas *Ulmus pumila* and its cultivars presented distinct temperature discrepancies within the Deciduous Tree group. These variations in T_canopy_ across different species and functional types are likely closely correlated with species-specific attributes, including stomatal conductance, leaf morphology, and canopy structural characteristics.

**Fig 4:**
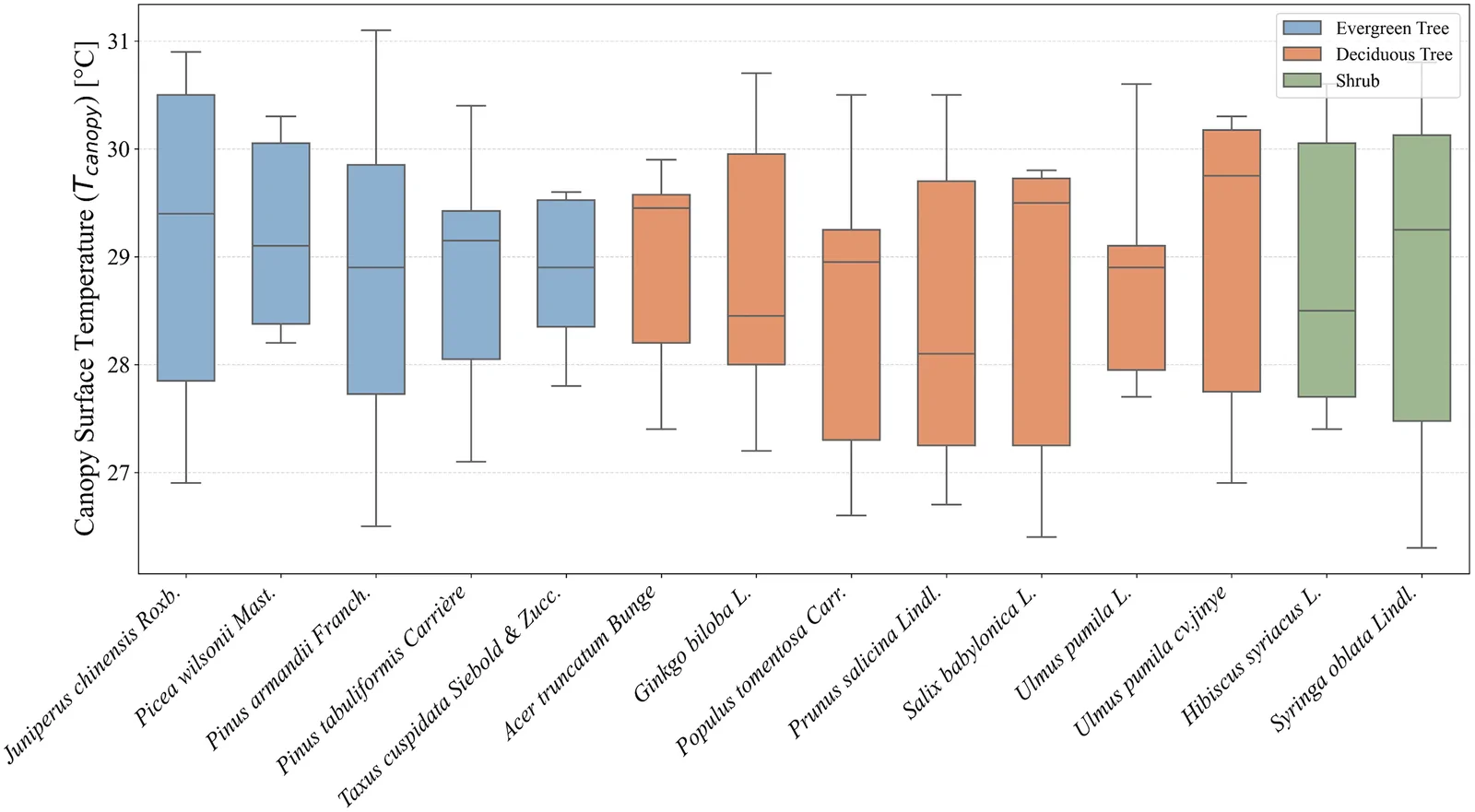
Interspecific variation of canopy surface temperature (T_canopy_) across 14 plant species within three functional types.

To further investigate the driving mechanisms associated with plant physiological thermal status and microclimate, a regression analysis was performed between canopy surface temperature (T_canopy_) and the magnitude of thermal comfort improvement (ΔPET) (Fig 5). Results indicate that across the three Plant Functional Types, no significant linear correlation was exhibited between ΔPET and T_canopy_ (R^2^ ≤ 0.09, P > 0.05). Although the fitted lines presented a weak trend, the statistical non-significance effectively counters the hypothesis positing the singular dominance of “surface long-wave radiative cooling.” This finding reveals that the amelioration of sub-canopy PET by vegetation relies predominantly on the direct interception of solar radiation by canopy structures (specifically, the reduction of mean radiant temperature, T_mrt_), rather than relying solely on the reduction of leaf surface temperatures.

**Fig 5:**
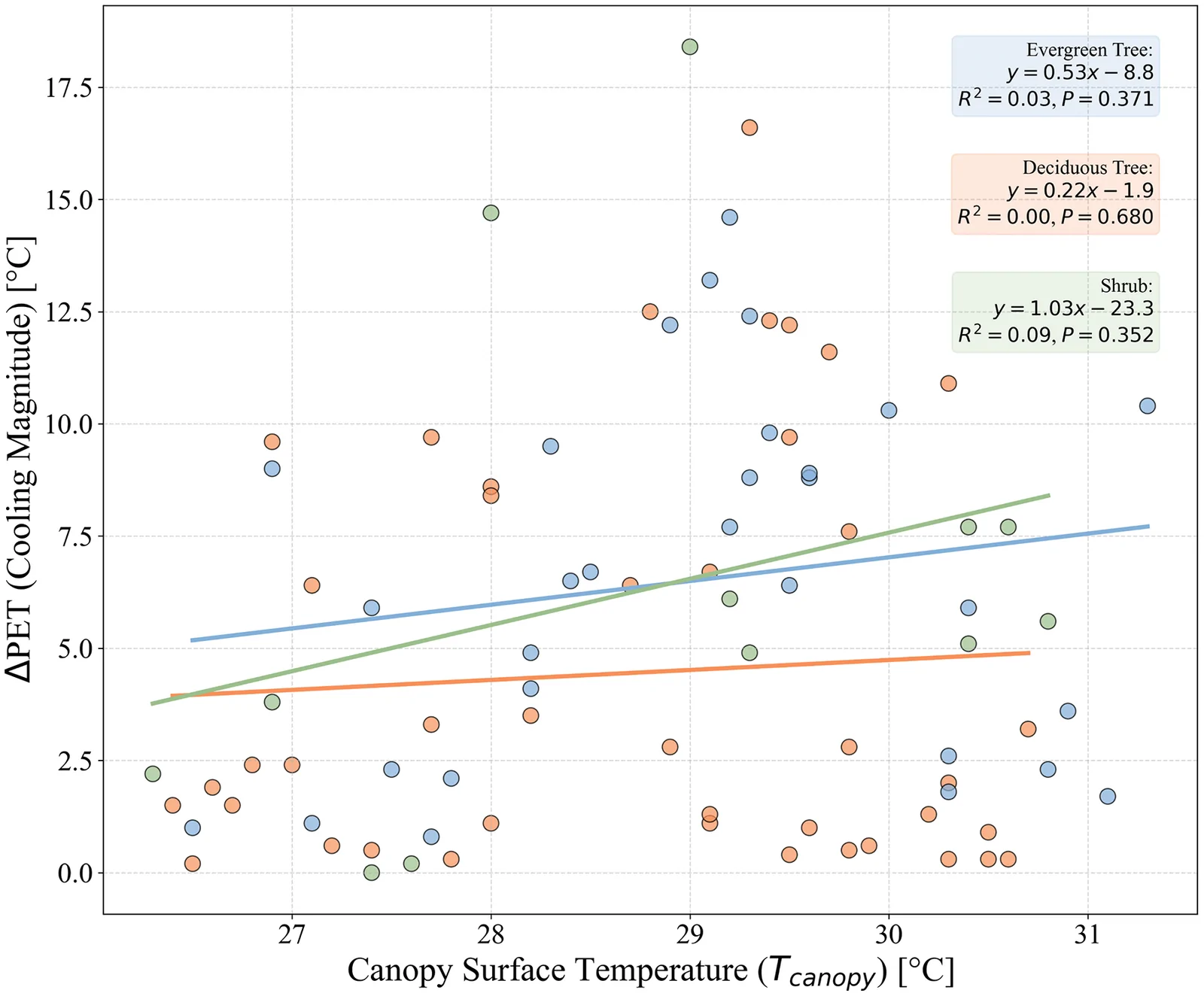
Relationship between canopy thermal state (T_canopy_) and microclimatic cooling magnitude (ΔPET).

Furthermore, the relationship between canopy surface temperature (T_canopy_) and the leaf-to-air temperature difference (ΔT_lc-a_) exhibits a comparable statistical “decoupling” phenomenon (R^2^ < 0.1, P > 0.35, Fig 6). This independence indicates that lower absolute values of canopy temperature do not necessarily equate to greater cooling efficiency. Synthesizing this with preceding analyses, variability in ΔT_lc-a_ is predominantly driven by the ambient background temperature (T_sun_) (manifested as high temperatures stimulating intense transpiration) rather than being determined by the absolute canopy temperature itself. This finding embodies a passive response mechanism utilized by plants to regulate cooling efficiency within dynamic thermal environments.

**Fig 6:**
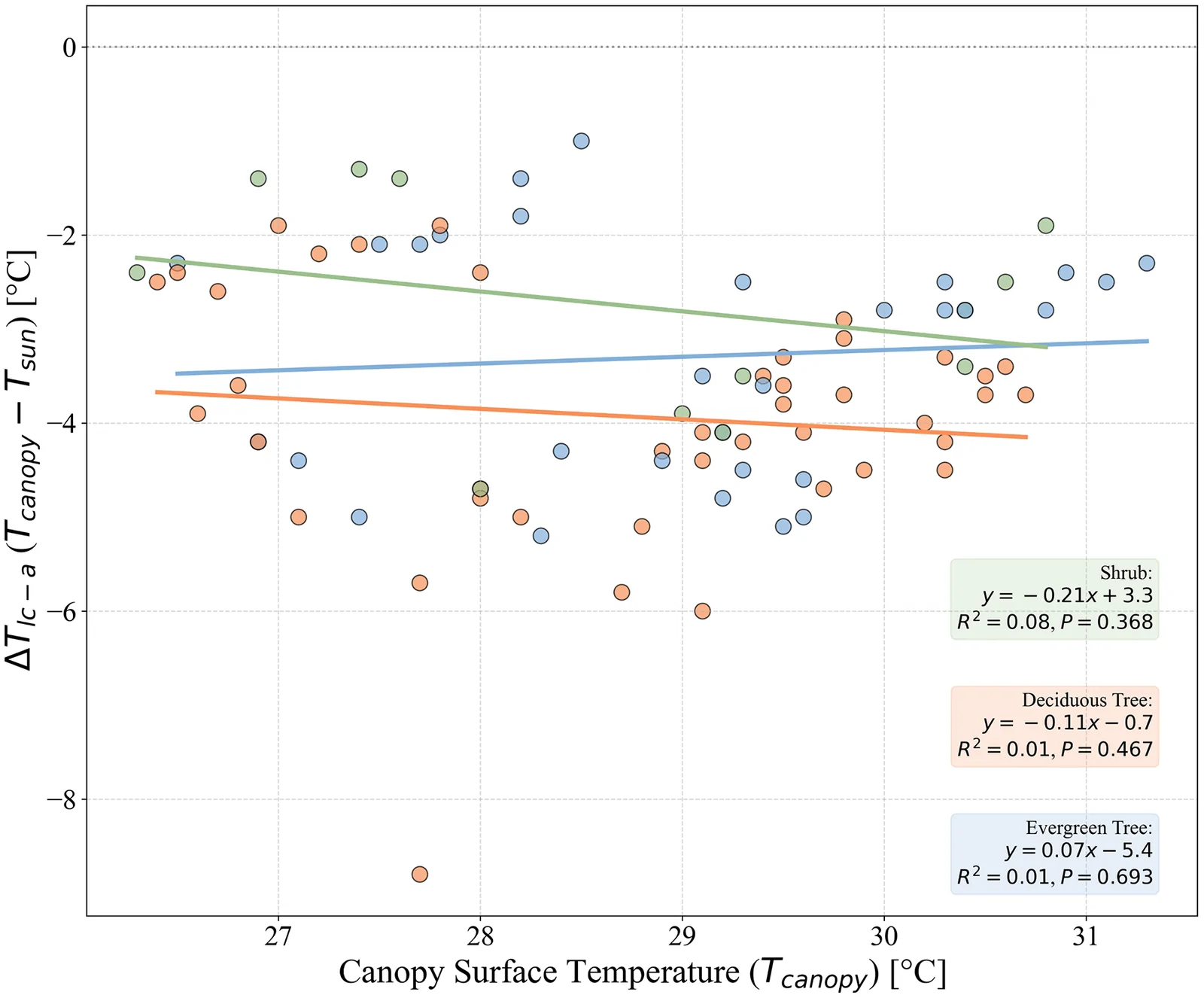
Correlation assessment of canopy thermal state (T_canopy_) versus physiological cooling differential (ΔT_lc-a_).

#### 3.2.2. Dominant mechanism of radiation blocking: Interaction network between structural parameters (LAI/SVF) and microclimate.

This study further examined the direct driving effects of canopy structural parameters (LAI and SVF) on thermal regulation indices to validate the traditional “structure-function coupling” hypothesis. This hypothesis postulates that a high Leaf Area Index (LAI) and a low Sky View Factor (SVF) should yield significant cooling benefits through enhanced transpiration and mechanical shading, respectively. However, results from the regression analysis revealed an unexpected characteristic of “Statistical Decoupling” (Fig 7).

**Fig 7:**
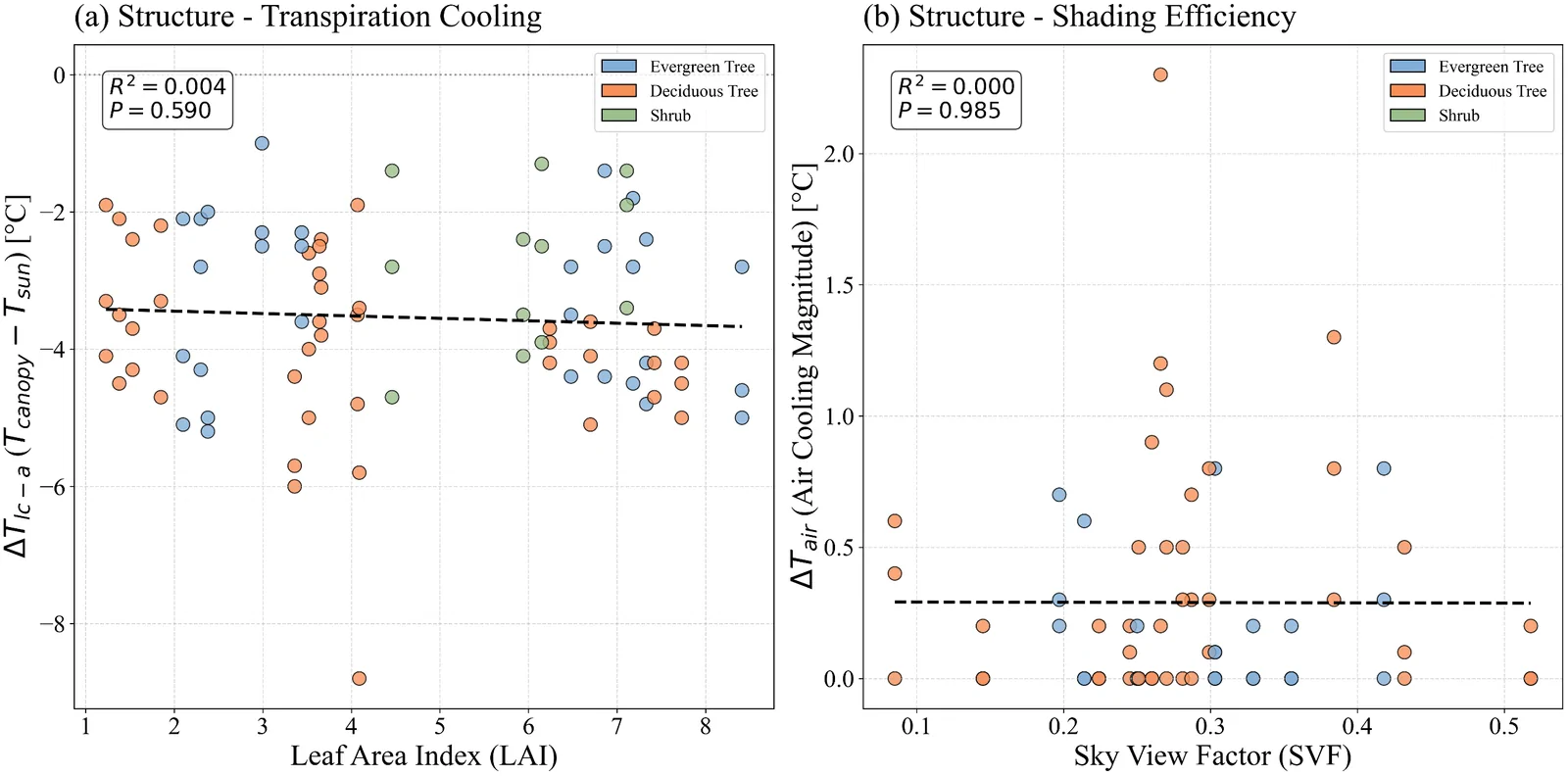
Linear regression analysis of canopy structural parameters versus transpiration cooling and shading efficiency.

Specifically, although Evergreen Trees possess a high mean LAI of 6.39, the increase in Leaf Area Index did not result in a statistically significant enhancement of the leaf-to-air temperature difference (ΔT_lc-a_) (R^2^ = 0.004, P = 0.590). This counter-intuitive result strongly implies that the transpiration cooling efficiency of plants is governed predominantly by species-specific physiological regulatory mechanisms (such as stomatal conductance and sensitivity to VPD) rather than relying solely on the physical accumulation of leaf quantity. In other words, merely increasing the transpiration surface area (LAI) without the accompaniment of efficient stomatal regulation cannot guarantee a stronger conversion of latent heat flux.

Similarly, no significant linear correlation was exhibited between the Sky View Factor (SVF) and the magnitude of air cooling (ΔT_air_) (R^2^ ≈ 0.000, P = 0.985). This indicates that within open and complex urban street canyon environments, the turbulent mixing and airflow effects may largely “dilute” or attenuate the local radiative cooling effects induced by canopy shading (reduced SVF). Consequently, singular geometric structural parameters are insufficient to explain the variability in microclimate cooling. Future urban greening configurations must shift from mere “structural accumulation” toward a comprehensive consideration of “physio-physical” coupling mechanisms.

The comprehensive Pearson correlation matrix presented in Fig 8 delineates a complex network of interactions among canopy structure, microclimatic factors, and thermal comfort indices. Initially, at the interface between structure and environment, canopy structural parameters demonstrated a significant radiative regulatory “leverage effect.” Specifically, the Leaf Area Index (LAI) exhibited a significant inverse correlation with the Sky View Factor (SVF) (r = −0.54, P < 0.001). This confirms that high-density vegetation reduces the load of downward short-wave radiation primarily by physically obstructing the view of the sky (thereby lowering SVF) rather than relying merely on structural accumulation to alter the thermal state of the canopy itself.

**Fig 8:**
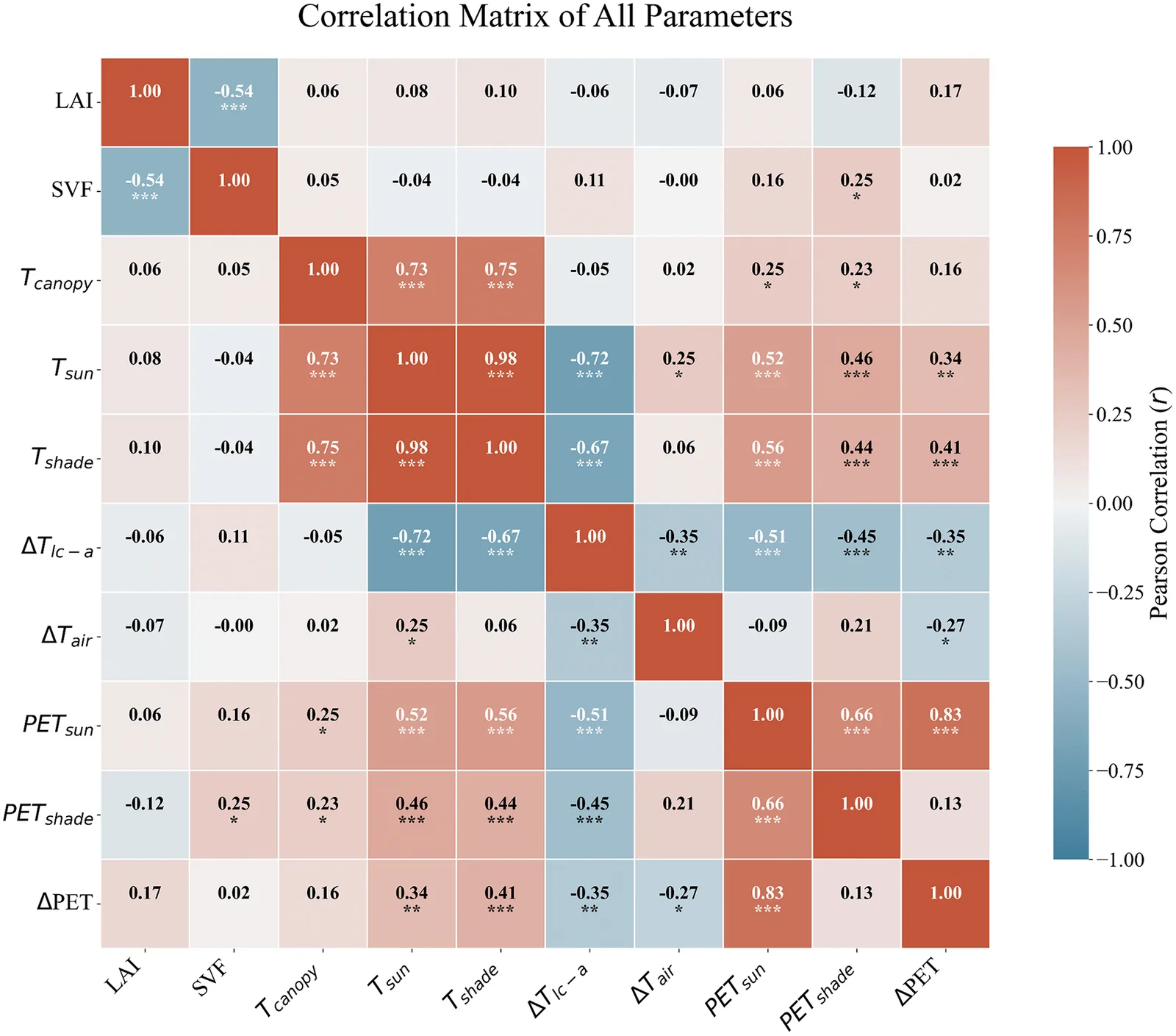
Heatmap of correlation coefficients illustrating the interactions between plant attributes and thermal environmental variables. Note: The numbers in the cells represent Pearson correlation coefficients (r). The color scale indicates the strength and direction of the correlation: red for positive and blue for negative. Asterisks denote statistical significance levels: * P < 0.05, ** P < 0.01, and *** P < 0.001.

Regarding the physiological thermal response of plants, canopy surface temperature (T_canopy_) displayed a robust positive coupling relationship with ambient air temperatures (T_sun_ and T_shade_) (r > 0.73, P < 0.001). While this indicates that canopy thermal conditions are highly dependent on the local microclimatic background, plants are not merely passive responders to their environment. Correlation analysis uncovered a critical “Stress-Cooling Mechanism.” The leaf-to-air temperature difference (ΔT_lc-a_) showed a significant inverse correlation with ambient air temperature (T_sun_) (r = −0.72, P < 0.001). This implies that as environmental heat stress escalates, the negative magnitude of ΔT_lc-a_ increases significantly. Such a trend indicates that under conditions of high temperature stress, plants tend to maintain physiological homeostasis by enhancing transpiration. *Ulmus pumila* within the Deciduous Tree group exemplifies this mechanism. Despite being subjected to the highest environmental heat pressure within the sample (where T_sun_ reached 34.5°C), it maintained an extreme leaf-to-air temperature difference of −5.7°C. This demonstrates an adaptive strategy to extreme environments whereby the impact of high temperatures is offset by vigorous transpiration efficiency.

Finally, the matrix heat map elucidated the critical pathways for ameliorating the outdoor thermal environment during summer. An extremely strong positive correlation exists among solar radiation (G_sun_), globe temperature (Tg), and the human thermal comfort index (PET) (r > 0.9, P < 0.001). This confirms that short-wave radiative load is the primary physical factor driving thermal discomfort in summer. Although relative humidity (RH) and wind speed exert a certain regulatory effect on PET, their correlation coefficients are significantly lower than those of radiation parameters. This result offers vital design implications. Specifically, under high-radiation weather conditions during summer in Temperate Monsoon Climate Region, the prioritization of radiative blocking (Shading) through vegetation configuration holds a weight far superior to that of ventilation and heat dissipation. Consequently, it constitutes the paramount strategy for improving outdoor thermal comfort on campuses. The high determination coefficient (R² > 0.85) between Tmrt and PET suggests more than a simple correlation; it reflects a causal energy pathway where canopy-induced shading acts as the primary filter for short-wave radiation. Our step-wise regression confirms that variations in the radiative heat exchange account for over 70% of the PET variance, effectively overshadowing the secondary cooling effects of latent heat from transpiration in these high-density canyons.

To further elucidate the physical pathway through which canopy structure improves thermal comfort, the linear correlations between Leaf Area Index (LAI), the reduction in solar radiation (ΔSR), and the improvement in PET (ΔPET) were analyzed. As shown in Table 3, a significant positive correlation was observed between LAI and ΔSR (r = 0.219, p = 0.045 < 0.05), confirming that denser canopies (higher LAI) effectively intercept more solar radiation. Furthermore, a strong positive correlation was identified between ΔSR and ΔPET (r = 0.659, p < 0.001), indicating that the attenuation of solar radiation is the primary driver for PET reduction. These results illustrate a clear causal chain: higher LAI enhances radiation shielding (ΔSR), which in turn significantly improves human thermal comfort (ΔPET), even if the air temperature reduction is limited.

**Table 3:** The correlation between ΔPET, LAI and ΔSR.

Relationships	Pearson Correlation (r)	Sig. (2-tailed)	N	Description
**LAI vs.ΔSR**	0.219*	0.045	84	Positive correlation (Significant at 0.05 level)
**ΔSR vs.ΔPET**	0.659**	0	84	Strong positive correlation (Significant at 0.01 level)

Note: ΔSR: Difference in solar radiation between sunlight and shade; ΔPET: The difference in physiological equivalent temperature between sunlight and shade.

* Indicates significance at P < 0.05, ** indicates significance at P < 0.01.

### 3.3. Mechanism-based spatial optimization strategies and simulation assessment

Mechanistic analysis has substantiated that “radiative blocking” and “blue-green synergy” constitute critical pathways. Grounded in these underlying mechanisms, this study developed multiple spatial optimization scenarios to further quantify the marginal efficacy of distinct intervention strategies via numerical simulation.

#### 3.3.1 Vegetation structure reconfiguration: Transitioning from “Green Volume Expansion” to “Qualitative Enhancement”.

To translate the microclimatic regulatory patterns identified through field measurements into actionable spatial optimization strategies, this study utilized the ENVI-met model calibrated using empirical data. This model demonstrated a Root Mean Square Error (RMSE) of 2.27°C for temperature and 8.96% RH for humidity. Optimization scenarios were formulated targeting vegetation structure, blue-green spatial synergy, and underlying surface materials to quantify the magnitude of thermal comfort (PET) improvement yielded by various intervention measures. In total, 12 scenarios were designed, covering four categories: single vegetation types (evergreen trees, shrubs, lawn), single water body / paving settings, and composite blue-green configurations. Detailed parameter settings and spatial layouts for each scenario are provided in S3 Table in the Supporting Information. Regarding the validation of the Physiological Equivalent Temperature (PET), although direct field measurements of PET were not performed, the reliability of the simulated PET results is supported by the high accuracy of the primary input parameters. As PET is a secondary thermodynamic index derived from air temperature (Ta), relative humidity (RH), wind speed (Va), and mean radiant temperature (Tmrt), the rigorous validation of Ta and RH in this study ensures the fundamental integrity of the calculation. Furthermore, the ENVI-met model has been extensively documented in previous literature as a robust tool for predicting radiant heat exchange and PET in high-density urban environments. By maintaining low RMSE values for the core meteorological variables, the resulting PET values are considered to be within a scientifically acceptable range for evaluating thermal comfort trends across the campus.

Simulation results presented in Table 4 forcefully substantiate the pivotal role of mature arborous vegetation in ameliorating thermal comfort. Upon replacing existing vegetation with evergreen trees reaching a height of 25 m (Scenario 1−1), the mean daily PET descended significantly to 48.9°C. In stark contrast, scenarios featuring exclusively shrub planting (Scenario 1−2, with a PET of 55.1°C) or turf grass (Scenario 2−1, with a PET of 55.3°C) yielded negligible improvements to the thermal environment. Consequently, the differential in ΔPET between evergreen trees and the shrub or turf layers reached a magnitude ranging from 6.2°C to 6.4°C. This substantial disparity in efficacy indicates that merely increasing green coverage rates, such as through extensive lawns, is insufficient to mitigate heat stress. Instead, “vertical spatial radiative blocking” provided by tall trees constitutes the definitive “quality” metric for microclimatic enhancement.

**Table 4:** Statistical summary of air temperature, relative humidity, and PET under different simulation scenarios.

Scenario	Ta (℃)			RH (%)			PET (℃)		
	MAX	MIN	AVERAGE	MAX	MIN	AVERAGE	MAX	MIN	AVERAGE
**1−1**	31	23	29.1	86.7	56.4	63.9	57.3	31.7	48.9
**1-2**	31.5	27.5	30	74	54.9	60.9	57.6	41	55.1
**2−1**	31.6	27.4	30	73.8	55.1	60.9	57.6	42.4	55.3
**3−1**	31.1	27.4	29.9	73.9	54.6	60.9	57.6	42	55.2
**3−2**	31.3	27.1	29.8	74	55	61.6	57.5	38.4	49.6
**4−1**	31.6	27.5	30	70.4	54.6	60	57.6	42.6	54.9
**4−2**	31.7	27.8	30.4	69.6	54	59	57.7	42.6	55.4
**5−1**	31	26.1	29.2	86.6	56.4	63.9	57.4	34.4	48.9
**6−1**	30.9	23.2	29	86.5	56.5	64.3	57.4	31.1	48.7
**7−1**	22.9	31	29.1	85.2	56.1	63.3	57.3	31.7	48.8
**7−2**	23.6	31.1	29.3	82.1	55.7	62.5	57.4	31.8	49

#### 3.3.2. Synergistic blue-green spaces: The superimposed effect of dual cooling modes.

The intervention of water bodies exhibits distinct scale effects and synergistic potential regarding the modification of the thermal environment. In comparison with the maintenance of existing water bodies (Scenario 3−1, with a PET of 55.2°C), the expansion of water surface area combined with the incorporation of dynamic forms (Scenario 3−2) resulted in a precipitous decline in mean daily PET to 49.6°C. This represents a reduction magnitude of 5.6°C. Of particular note is the synergistic mode combining trees and water bodies (Scenario 6−1). When tall evergreen trees were integrated with preserved water features, the PET decreased further to 48.7°C, thereby emerging as the optimal solution among all simulated scenarios. This “dual-source cooling” mechanism—characterized by the interception of radiation via arborous shading and the absorption of latent heat through aqueous evaporation—demonstrates the additive advantages of blue-green infrastructure in mitigating extreme heat.

#### 3.3.3. Subsurface material fine-tuning: Limitations of passive adjustment.

Relative to vegetation and water bodies, the substitution of underlying surface materials makes a comparatively marginal direct contribution to thermal comfort. Under conditions devoid of shading, granite paving (Scenario 4−1, with a PET of 54.9°C) yielded a reduction of merely 0.5°C in comparison to wooden decking (Scenario 4−2, with a PET of 55.4°C). Conversely, beneath the canopy of trees (comparing Scenario 7−1 and Scenario 7−2), the PET variance attributable to distinct paving materials becomes virtually negligible (less than 0.2°C). This evidence suggests that within hardscape design, the selection of paving materials holds a weight far inferior to that of upper shading structures. Consequently, the maxim of “Shade First, Paving Second” should be adopted as a priority design principle.

## 4. Discussion

### 4.1. Differences in plant functional types and thermal regulation mechanisms of vertical structures

The empirical ranking of cooling efficacy derived from this study—specifically, Evergreen Trees (ΔPET 10.6°C) ≈ Shrubs (10.4°C)> Deciduous Trees (8.1°C)—rectifies the traditional cognition that predicts cooling capacity based solely on biomass. Instead, it reveals the determinative role of vertical spatial radiative blocking efficiency in microclimate regulation [60].

The dominant position of Evergreen Trees stems from their structural characteristics defined by “high canopy density and low SVF.” As a primary physical barrier, this dense needle canopy effectively intercepts the vast majority of incident solar short-wave radiation (G_sun_) [61]. SVF represents a critical microclimatic indicator denoting the visible extent of the sky at a specific point; a low SVF implies heightened shading, thereby reducing solar radiation [62]. However, a more critical discovery pertains to the high efficacy of the shrub layer. Results indicate that although shrubs lack the canopy height of trees, their morphology of “dense growth proximate to the ground” constructs an extremely efficient radiation protection layer within the sensitive height range of human core thermal perception (extending from 0.5 m to 1.5 m) [63]. Synthesizing this with the extremely strong coupling relationship between radiation parameters and PET observed in the correlation analysis (Table 5 and Table 6, where r > 0.9), it becomes evident that human thermal comfort is driven primarily by the mean radiant temperature (T_mrt_) [61]. Shrub thickets not only effectively sever the heat exchange pathway between terrestrial long-wave radiation and the human body but also suppress sensible heat flux through near-surface transpiration. Consequently, they exhibit characteristics superior to deciduous trees, which possess sparse canopies and elevated branching points, regarding local microclimate improvement [60,64,65].

**Table 5:** Pearson correlation analysis between Physiological Equivalent Temperature (PET) and meteorological parameters under direct sun.

		Ta_sun_	RH_sun_	Va_sun_	MRT_sun_
**PET**_**sun**_	Pearson Correlation	0.524**	−0.704**	0.41**	0.916**
	Sig. (2-tailed)	0.000	0.000	0.000	0.000
	N	84	84	84	84

**Table 6:** Pearson correlation analysis between Physiological Equivalent Temperature (PET) and meteorological parameters under tree shade conditions.

		Ta_shadow_	RH_shadow_	Va_shadow_	MRT_shadow_
**PET**_**shadow**_	Pearson Correlation	0.433**	−0.633**	0.309**	0.765**
	Sig. (2-tailed)	0.000	0.000	0.000	0.000
	N	84	84	84	84

Notably, the analysis of cooling efficacy based on landscape element categories (Fig 5) also unveiled a critical statistical characteristic: in comparison to the relatively short error bars observed for Artificial Shade Devices and Tree Groves, the Evergreen Tree and Shrub groups exhibited extremely large standard deviations. This significant intra-group variability strongly implies the existence of “interspecific heterogeneity.” In other words, not all evergreen trees or shrubs possess identical cooling capacities [37,40]. Instead, their specific efficacy is highly dependent on species-specific attributes such as Leaf Area Index (LAI), canopy geometry, and the height of branching points [52,60,61,64–66]. This evidence further corroborates that within landscape design, merely increasing green volume is insufficient. Rather, the selection of specific species characterized by high shading efficiency (such as Picea asperata, which demonstrated superior performance in this study) constitutes the key to enhancing thermal comfort [67,68].

### 4.2. Statistical decoupling of “Structure-Physiology” and the mechanism of physical dominance

The traditional “Structure-Function Coupling” hypothesis postulates that a higher Leaf Area Index (LAI) should yield more significant cooling effects through enhanced transpiration [66]; however, the regression analysis in this study (Fig 7) reveals a significant characteristic of “Statistical Decoupling” [60].

Initially, no significant linear correlation was exhibited between LAI and the leaf-to-air temperature difference (ΔT_lc-a_) (P = 0.590). This result effectively counters the simplistic deduction that “the accumulation of leaf quantity automatically induces transpiration cooling” [37,60]. Instead, it indicates that the active regulation efficiency of plants is governed predominantly by species-specific stomatal physiological mechanisms (such as sensitivity to VPD and stomatal conductance) rather than relying solely on morphological structural parameters [69,70].

Secondly, the non-significant correlation between the Sky View Factor (SVF) and the magnitude of air cooling (ΔT_air_) (P = 0.985) further suggests that within open campus environments, the effects of turbulent mixing substantially attenuate the reduction in air temperature induced by local shading [60,70].

In summary, the mechanisms of microclimatic regulation exhibit a distinct characteristic of “physical radiative dominance.” In comparison to unstable air cooling (ΔT_air_) or transpiration cooling contingent upon physiological states (ΔT_lc-a_), the direct interception of radiative input via the reduction of SVF constitutes the most immediate and reliable pathway for ameliorating human thermal comfort (PET) [11,61,62]. This elucidates why certain plants can still achieve superior improvements in thermal comfort, provided their physical structures are sufficiently dense (characterized by low SVF), even if their physiological cooling capacities are limited [60]. The observed divergence between LAI and physiological cooling suggests a “statistical decoupling” under extreme thermal stress. This indicates that radiative shielding (physical blocking) by the canopy architecture, rather than evapotranspiration (physiological regulation), becomes the dominant determinant of PET mitigation in open urban canyons. This finding necessitates a shift from “biomass-centric” to “structure-centric” cool island design. The findings provide empirical and simulative evidence that radiative shielding is the mechanistic driver of thermal relief in the studied campus morphology. While convective and evaporative cooling contribute to air temperature reduction, the modulation of the long-wave and short-wave radiation balance by the urban canopy is the dominant physical determinant in optimizing individual human energy budgets during peak heat hours.

### 4.3. The dual role of canopy temperature: A trade-off between radiative barrier and secondary heat source

This study confirms that canopy surface temperature (T_canopy_) is not a singular linear factor determining sub-canopy thermal comfort; rather, the relationship between the two exhibits a complex non-linear character (manifested as statistical decoupling, where P > 0.05) [60]. This finding unveils a trade-off regarding the dual roles played by vegetation in thermal regulation: functioning simultaneously as a “radiative barrier” and a “secondary heat source” [71,72].

On one hand, while Evergreen Trees (such as *Picea wilsonii*) provide maximum shading benefits [73,74], certain species like *Juniperus chinensis* maintain elevated canopy surface temperatures (reaching 29.1°C) due to obstructed heat dissipation caused by leaf morphological characteristics. Consequently, the heated canopy itself may transform into a source of downward long-wave radiation. This phenomenon partially offsets the cooling dividend derived from shading [60] and results in a surge in PET values in sun-exposed areas (reaching 49.9°C).

On the other hand, although some Deciduous Trees (such as *Salix babylonica*) possess a shading density inferior to that of evergreens, their higher stomatal conductance and transpiration efficiency enable them to maintain lower leaf temperatures (approximately 28.5°C). This effectively minimizes the emission of secondary thermal radiation [73–75].

Therefore, the magnitude of ΔPET improvement is fundamentally the result of a contest between “short-wave radiative blocking (positive benefit)” and “canopy long-wave radiative emission (negative benefit)” [60]. The finding that Evergreen Trees achieved the optimal overall ΔPET (10.6°C) indicates that under summer conditions of high radiation, the weight of “blocking solar short-wave radiation” far exceeds the negative impact of “canopy self-heating” [73,76]. However, in scenarios characterized by extreme heat or poor ventilation, the selection of “dual-optimal” species—those capable of providing both high-density shading (low SVF) and maintaining low leaf temperatures through vigorous transpiration (low T_canopy_), such as the Picea asperata identified in this study—will be critical for maximizing microclimatic benefits [61,73].

### 4.4. “Stress-Cooling” adaptive strategies under high-temperature stress

This study establishes a “Hierarchy of Radiative Blocking” for temperate campus landscapes. We propose an asymmetrical planting model (deciduous for solar access in winter, evergreen for radiative defense in summer) to resolve the seasonal microclimate paradox. Furthermore, the identified optimal blue-green coupling ratio serves as a quantitative threshold for maximizing latent heat absorption without compromising ventilation. The significant inverse correlation between the leaf-to-air temperature difference (ΔT_lc-a_) and ambient air temperature (T_sun_) (r = −0.72), as revealed by correlation analysis, characterizes the “Stress-Cooling Mechanism” employed by plants to cope with high-temperature stress. As environmental heat stress escalates, plants do not passively undergo heating; instead, they maintain physiological homeostasis by significantly amplifying the leaf-to-air temperature difference (specifically, by increasing the magnitude of negative values) [60]. Taking *Ulmus pumila* as an illustrative example, this species maintained an extreme leaf-to-air temperature difference of −5.7°C while simultaneously enduring the highest environmental heat load of 34.5°C. This non-linear response, which follows the pattern that “hotter environments trigger stronger cooling,” demonstrates that under conditions characterized by adequate water availability, plants are capable of prioritizing survival advantages at the expense of water consumption [60]. Although the direct contribution of this active regulatory mechanism to sub-canopy PET is less pronounced than that of physical shading (owing to the attenuation caused by air mixing effects), it holds profound biophysical significance for lowering the heat load of the canopy itself and minimizing the downward emission of long-wave radiation. Consequently, it serves as the physiological foundation for constructing high-resilience campus ecosystems [60].

#### 4.4.1. Practice-oriented approach: design strategies for enhancing campus thermal comfort within temperate monsoon climate regions.

Based on a comprehensive analysis integrating empirical mechanisms and simulation optimizations, this study proposes a “hierarchical” design strategy tailored for the improvement of thermal environments in summer outdoor spaces within temperate monsoon climate regions. The objective of this strategy is to provide guidance for campus landscape renewal practices [38,45,76,77].

#### 4.4.2. First priority: Establishing a core shading network centered on evergreen trees.

Simulation results clearly defined the hierarchy of efficacy as “Evergreen Trees exceeding Shrubs, which in turn exceed Lawns” [60,73,76]. Consequently, it is recommended to prioritize the construction of a shading skeleton centered on high-density evergreen trees (such as *Picea wilsonii* and *Juniperus chinensis*) along campus main roads, at plaza edges, and within areas of high-frequency activity [73,74,78]. Evergreen species not only provide a ΔPET reduction reaching as high as 10.6°C during summer (supported by empirical data) but also demonstrated high compatibility with underlying surface materials in simulations. This means they maintain low PET levels regardless of whether the underlying surface consists of turf or paving [73,76]. Addressing the requirements for winter daylighting in temperate monsoon climate zones, it is advisable to adopt an asymmetric configuration pattern characterized by “deciduous species on the south and evergreen species on the north.” Specifically, deciduous trees with elevated branching points should be planted on the southern side to permit the penetration of warm winter sunlight, while evergreen trees should be densely planted on the northern and western sides to obstruct cold winter winds while providing intense shading during summer [60,79,80].

#### 4.4.3. Second priority: Activating the dual cooling potential of “Evaporation and Visual Perception” regarding water bodies.

The cooling potential of 5.6°C demonstrated by simulations proves that water bodies serve as a key regulatory factor second only to arborous vegetation [76]. Design strategies should avoid reliance solely on static water surfaces, shifting instead toward strategies combining expanded water surface areas with dynamic forms such as fountains and cascades. Furthermore, these features should be positioned in areas upwind of the dominant summer wind direction (typically the southwest) to utilize the “cold advection” effect for expanding the cooling range [76]. Additionally, water bodies should be integrated with arboreal shading to the greatest extent possible (following the mode of Scenario 6−1). This is essential to prevent open water surfaces from absorbing excessive radiation at noon and subsequently becoming secondary heat sources, thereby realizing the optimal cooling benefits of “forest-water symbiosis” [75,76].

#### 4.4.4. Third priority: Auxiliary regulation via permeable cool materials.

Although the direct contribution of paving materials to cooling is limited [34], material selection remains critical within open gathering plazas where arbor planting is unfeasible. It is recommended to comprehensively replace high-heat-absorbing dark asphalt with light-colored granite or permeable bricks characterized by high albedo (exceeding 0.3) and high permeability [34,71]. This approach not only fine-tunes the thermal environment by reducing surface long-wave radiation (reducing PET by approximately 1.9°C) but also enhances soil water retention capabilities through rainwater infiltration. Consequently, this indirectly supports the transpiration cooling functions of surrounding vegetation, achieving dual benefits for both ecology and microclimate [81–83]. The data indicates that increasing the proportion of evergreen species could be a viable strategy for mitigating summer PET in similar high-density urban canyons. However, the implementation of such strategies should be carefully weighed against other ecological factors, such as winter solar access and native biodiversity requirements.

### 4.5. Limitations and future prospects

Before addressing methodological limitations, we clarify the scientific motivation for studying summer heat stress in a temperate monsoon climate zone. While “temperate” may imply mild conditions, summer here features concurrent high temperature and high humidity—a “humid-heat” regime driven by East Asian monsoon moisture transport. Under global warming, sustained high humidity intensifies heat stress even without extreme temperature rises. Thus, mitigating outdoor discomfort here requires strategies addressing both radiative and convective loads, and our use of PET—integrating temperature, humidity, wind, and radiation—is particularly appropriate for this context.

Regarding temporal representativeness, we acknowledge that field observations were limited to a single day (July 11, 2024). While these conditions align with July climatological normals for Shenyang, one-day data cannot capture the full spectrum of summer weather variability (e.g., different heatwave durations, cloud cover, or antecedent rainfall) nor day-to-day soil moisture fluctuations affecting transpiration. To mitigate this, we adopted a hybrid approach: field measurements served primarily for model calibration and validation, while the validated ENVI-met model explored multiple spatial scenarios under repeatable boundary conditions, compensating for the temporal sparsity of observations.

Beyond temporal scope, other limitations warrant acknowledgment. This study examined individual landscape elements rather than complex multi-layered communities (e.g., tree-shrub-grass or mixed evergreen-deciduous forests) and lacked quantitative weighting among elements. The evaluation relied solely on PET without direct validation of mean radiant temperature, nor did it account for behavioral characteristics or subjective thermal perception. Spatial scope is limited to a single campus in cold-region China, and the sample size of 14 species constrains the generalizability of the “structural-thermal decoupling” effect.

Future research should extend to full annual cycles with multi-season monitoring to capture seasonal trade-offs (summer shading vs. winter solar access), elevate spatial scales from individuals to communities, integrate behavioral data to construct “microclimate-behavior-perception” models, and expand to broader climatic zones and urban typologies. Addressing the “Dual Carbon” goals, future work should also explore synergy between green infrastructure (e.g., permeable pavements, rain gardens) and microclimate regulation. Accordingly, the numerical effect sizes, element rankings, and configuration thresholds reported here should be interpreted as conditional on the sampled campus morphology, the species set examined, and the meteorological boundary conditions used for model calibration and scenario simulation.

## 5. Conclusions

Based on field observations conducted on one representative summer day at a single university campus in Shenyang and on ENVI-met simulations calibrated against those observations, this study draws context-specific conclusions for the examined campus morphology and meteorological boundary conditions. The findings indicate how selected landscape elements and their combinations may regulate PET during extreme summer heat in a temperate monsoon climate; however, the reported effect sizes, rankings, and spatial thresholds should not be assumed to apply directly to other campuses, urban forms, seasons, or weather conditions. Multi-site and multi-season validation is required before broader generalization. Within these boundaries, the following key conclusions were obtained:

(1) Dominance of Radiative Blocking in Vertical Structures: Empirical data confirmed a cooling hierarchy where Evergreen Trees ≈ Shrubs > Deciduous Trees. Evergreen trees, distinguished by a high Leaf Area Index (LAI = 6.39) and low Sky View Factor (SVF), reduced midday PET (12:00–14:00) by 10.6°C, establishing their status as core “cool sources.” Notably, shrubs created efficient near-surface radiation barriers through dense foliage, achieving a mean ΔPET of 10.4°C, which is statistically superior to that of deciduous trees (8.1°C). This finding corrects reliability on biomass-based predictions, emphasizing that radiative blocking efficiency within the core thermal perception range (extending from 0.5 m to 1.5 m) is decisive for thermal comfort.
(2) Optimal Spatial Configuration of “Blue-Green Synergy”: Multi-scenario simulations revealed non-linear superposition effects of landscape elements. The synergistic mode combining “tall evergreen trees and dynamic water” (Scenario 6−1) exhibited the highest thermal regulation potential, reducing the daily mean PET to 48.7°C (a 7.3°C decrease from the baseline). This efficacy significantly outperforms solitary water expansion (Scenario 3−2, 49.6°C) or solitary evergreen planting (Scenario 1−1, 48.9°C), confirming a positive synergistic mechanism between “short-wave radiation interception by trees” and “latent heat absorption via water evaporation.” Furthermore, dynamic water features provided an additional PET improvement of 3.1°C compared to static water.
(3) Auxiliary Regulation and Thresholds of Underlying Surfaces: Compared to biotic elements, abiotic surfaces play a secondary role characterized by strong “shade dependency.” Under conditions devoid of shading, high-albedo granite paving reduced PET by 1.9°C compared to dark asphalt, demonstrating potential for heat stress mitigation. However, under tree canopy shading, the PET difference induced by varying paving materials was merely 0.2°C. Consequently, prioritizing the construction of upper shading skeletons is paramount, while “Cool Pavement” strategies should be applied primarily to open gathering plazas where arbor planting is unfeasible.
(4) Hierarchical Strategies for Thermal Comfort Enhancement: Integrating empirical and simulation results, this study proposes a tiered strategy: First, establish a backbone shading network centered on high-density evergreen species (e.g., *Juniperus chinensis*, *Picea asperata*), adopting an asymmetric “Deciduous South (for winter light), Evergreen North (for winter wind protection and summer shading)” pattern. Second, activate blue-green cool islands by positioning “tree and fountain” composite landscapes upwind of dominant summer winds to utilize cold advection effects. Finally, optimize micro-morphology by prioritizing enclosed pavilions (offering a ΔPET improvement of 2.9°C, superior to permeable pergolas) to maximize radiative blocking benefits.

Within the specific campus and summer conditions examined, this study provides context-dependent evidence on the microclimatic performance of landscape elements and offers testable design hypotheses for campuses in comparable temperate monsoon settings. Their transferability to other sites, urban morphologies, seasons, and meteorological conditions should be evaluated through additional field observations and cross-regional model validation.

## Supporting information

S1 Fig Measurement schematic. (TIF)

S1 Table Basic information of sample trees. (XLSX)

S2 Table Environmental characteristics and all-sky imagery of landscape element measurement points. (XLSX)

S3 Table Description and configuration of ENVI-met simulation scenarios for spatial optimization. (XLSX)
