# Supplementary material for: Microclimatic regulation effects of landscape elements on university campus in a temperate monsoon climate zone: A case study during extreme summer heat
Source: PLoS One. 2026 Nov 5;21(11):e0362099. doi: 10.1371/journal.pone.0362099 (PMC13739154; doi:10.1371/journal.pone.0362099)
Supplement: Supplementary file 1 [file pone.0362099.s007.docx]

Response to Reviewer Comments

Dear editor and reviewers:

We would like to thank you for your professional review work, constructive comments, and valuable suggestions on manuscript. Your time and efforts are greatly our appreciated. We have strived to improve the manuscript accordingly as listed in details below. According to the Round 1 revision suggestions provided by editor and reviewers, all authors of this manuscript agreed to accept and adopt all the suggestions after discussion, and made the following responses to the valuable suggestions provided by reviewers point by point.

Thanks very much for taking your time to review this manuscript. We really appreciate all your comments and suggestions! Please find our itemized responses in below and my revisions:

**▪ Reviewer #1’s Comment:**

**Point #1:** The limitations of the study and the scope of its generalization should be further strengthened. The core conclusions still largely depend on a single campus environment and specific summer weather conditions. It is recommended that the authors further clarify the contextual dependence and limitations of the research results in the conclusion section.

**Response #1:** We agree with the reviewer and appreciate this important suggestion. We have further strengthened the description of the study limitations and narrowed the scope of generalization in the revised manuscript. First, at the end of Section 4.5, we now explicitly state that the numerical effect sizes, element rankings, and configuration thresholds are conditional on the sampled campus morphology, the examined species set, and the meteorological boundary conditions used for model calibration and scenario simulation. Second, we revised the opening paragraph of Section 5 to specify that the conclusions are based on field observations from one representative summer day at a single university campus in Shenyang and on ENVI-met simulations calibrated against those observations. We also clarify that the reported effect sizes, rankings, and spatial thresholds should not be directly generalized to other campuses, urban forms, seasons, or weather conditions without further validation. Third, we replaced the former broad final statement with a context-dependent conclusion and explicitly call for additional field observations and cross-regional model validation. These revisions do not introduce new data or alter any numerical results.

**Location of revision 1:** Section 4.5, “Limitations and Future Prospects,” page 51, lines 902–911 (yellow-highlighted text at lines 908–911).

“Accordingly, the numerical effect sizes, element rankings, and configuration thresholds reported here should be interpreted as conditional on the sampled campus morphology, the species set examined, and the meteorological boundary conditions used for model calibration and scenario simulation.”

**Location of revision 2:** Section 5, “Conclusions,” pages 51–52, lines 914–923 (yellow-highlighted paragraph).

“Based on field observations conducted on one representative summer day at a single university campus in Shenyang and on ENVI-met simulations calibrated against those observations, this study draws context-specific conclusions for the examined campus morphology and meteorological boundary conditions. The findings indicate how selected landscape elements and their combinations may regulate PET during extreme summer heat in a temperate monsoon climate; however, the reported effect sizes, rankings, and spatial thresholds should not be assumed to apply directly to other campuses, urban forms, seasons, or weather conditions. Multi-site and multi-season validation is required before broader generalization. Within these boundaries, the following key conclusions were obtained:”

**Location of revision 3:** Section 5, “Conclusions,” pages 53–54, lines 960–965 (yellow-highlighted paragraph).

“Within the specific campus and summer conditions examined, this study provides context-dependent evidence on the microclimatic performance of landscape elements and offers testable design hypotheses for campuses in comparable temperate monsoon settings. Their transferability to other sites, urban morphologies, seasons, and meteorological conditions should be evaluated through additional field observations and cross-regional model validation.”

**▪ Reviewer #2:**

**Comment:** All comments have been addressed.

**Response:** We thank Reviewer #2 for confirming that all previous comments have been addressed. No additional revision request was raised by Reviewer #2 in this round.

We sincerely appreciate the Editor’s and Reviewers’ time and constructive guidance. We hope that the revised manuscript is now suitable for publication in PLOS ONE.

Sincerely,

Dr. Yan Zhou
