# Supplementary material for: Microclimatic regulation effects of landscape elements on university campus in a temperate monsoon climate zone: A case study during extreme summer heat
Source: PLoS One. 2026 Nov 5;21(11):e0362099. doi: 10.1371/journal.pone.0362099 (PMC13739154; doi:10.1371/journal.pone.0362099)
Supplement: Supplementary file 2 [file pone.0362099.s006.doc]

Response to Reviewer Comments

Dear editor and reviewers:

We would like to thank you for your professional review work, constructive comments, and valuable suggestions on manuscript. Your time and efforts are greatly our appreciated. We have strived to improve the manuscript accordingly as listed in details below. According to the Round 1 revision suggestions provided by editor and reviewers, all authors of this manuscript agreed to accept and adopt all the suggestions after discussion, and made the following responses to the valuable suggestions provided by reviewers point by point.

Thanks very much for taking your time to review this manuscript. We really appreciate all your comments and suggestions! Please find our itemized responses in below and my revisions:

**▪ Editor’s Comments :**

**Point #1:** Please ensure that your manuscript meets PLOS ONE's style requirements, including those for file naming. The PLOS ONE style templates can be found at

https://journals.plos.org/plosone/s/file?id=wjVg/PLOSOne_formatting_sample_main_body.pdf and

https://journals.plos.org/plosone/s/file?id=ba62/PLOSOne_formatting_sample_title_authors_affiliations.pdf

**Response#1:** We have carefully reviewed the PLOS ONE style templates and reformatted our manuscript accordingly. This includes adjustments to file naming, heading styles, reference formatting, and overall document structure to comply with journal guidelines.

**Point #2:** In your Methods section, please provide additional information regarding the permits you obtained for the work. Please ensure you have included the full name of the authority that approved the field site access and, if no permits were required, a brief statement explaining why.

**Response #2:** We have added the following statement to Section 2.1 (Methodology) in the revised manuscript (marked in yellow):

"The study was conducted within the public campus areas of Shenyang Agricultural University. Field site access was granted via oral permission from the Logistics Management Department of Shenyang Agricultural University. No specific permits were required as the study did not involve private land, protected areas, or endangered/protected species."

**Point #3:** Please note that PLOS One has specific guidelines on code sharing for submissions in which author-generated code underpins the findings in the manuscript. In these cases, we expect all author-generated code to be made available without restrictions upon publication of the work. Please review our guidelines at https://journals.plos.org/plosone/s/materials-and-software-sharing#loc-sharing-code and ensure that your code is shared in a way that follows best practice and facilitates reproducibility and reuse.

**Response #3:** Our study utilized ENVI-met (commercial software) for numerical simulations, and all model parameters are fully described in Table 1 and Section 2.3. The statistical analyses were performed using standard commercial software (SPSS 26.0, Microsoft Excel). No original custom code was generated that underlies the findings. Therefore, code sharing is not applicable to this manuscript.

**Point #4:** We note that Figure 1,2 and S2 in your submission contain map/satellite images which may be copyrighted. All PLOS content is published under the Creative Commons Attribution License (CC BY 4.0), which means that the manuscript, images, and Supporting Information files will be freely available online, and any third party is permitted to access, download, copy, distribute, and use these materials in any way, even commercially, with proper attribution. For these reasons, we cannot publish previously copyrighted maps or satellite images created using proprietary data, such as Google software (Google Maps, Street View, and Earth). For more information, see our copyright guidelines: http://journals.plos.org/plosone/s/licenses-and-copyright.

**Response #4:** We have removed the copyrighted map/satellite images (Figure 1, Figure 2, and S2 Figure) entirely from the manuscript, deleted all related in-text references, and confirmed that the study's clarity and integrity remain intact through the detailed text descriptions and remaining original figures (S1 Fig, S1 Table, S2 Table).

**Point #5:** Based solely on observations from a single day on July 11, 2024, the data is severely underrepresented.

**Response #5:** We have removed the single-day field observation data (July 11, 2024) from the manuscript and now rely exclusively on the validated ENVI-met numerical simulations to support our findings, thereby eliminating the concern regarding temporal underrepresentation.

**Point #6:** Some conclusions are overgeneralized based on limited data.

**Response #6:** We have added a reference to Table 6 in the main text. In Section 4.1 (Discussion), we now explicitly refer to Table 6 (previously Table 5 in the revised version) when discussing the correlation analysis between PET and meteorological parameters. Specifically, the text now reads: "Synthesizing this with the extremely strong coupling relationship between radiation parameters and PET observed in the correlation analysis (Table 6, where r > 0.9)..."

**Point #7:** In page13-14, model validation is insufficient, validated only: Ta (temperature)/RH (humidity), particularly lacking validation of PET or radiation-related parameters.

**Response #7:** We have carefully reviewed the reviewer comments. No specific previously published works were recommended for citation. However, we have added relevant citations where suggested by the substantive feedback (see responses to Reviewer #1 below).

**▪ Reviewer 1's comments :**

**Point #1:** 1. In line 31, the claimed “non-linear mechanisms” are not supported by appropriate analytical methods.

**Response #1:** We thank the reviewer for this important observation. We agree that the original manuscript did not sufficiently demonstrate non-linear analytical methods. In the revised manuscript, we have:

1. Removed the unsupported claim of "non-linear statistical associations" from the abstract and replaced it with more accurate phrasing: "quantitatively dissects the regulatory mechanisms" (line 31 in revised manuscript).
2. Added non-linear analytical approaches in the Results section. Specifically, we have:
   a) Included regression analysis with R² values to demonstrate the absence of linear relationships where expected (Section 3.2.2, Figures 7 and 10)
   b) Added a causal chain analysis (Table 2) showing that ΔSR (solar radiation reduction) mediates the relationship between LAI and ΔPET
   c) Discussed the "statistical decoupling" phenomenon (R² ≈ 0.000-0.004, P > 0.05) as evidence of non-linear dynamics between structural parameters and physiological cooling
3. Revised the terminology throughout to accurately reflect that we are documenting the absence of linear relationships (decoupling) rather than claiming to have modeled non-linear mechanisms.

**Point #2:** The novelty is overstated, and the study largely follows established methodological frameworks.

**Response #2:** We acknowledge the reviewer's concern and have substantially revised the manuscript to present our contributions more realistically. Specifically:

1. Modified the abstract and introduction to avoid overstatement. The revised abstract now focuses on the specific findings for temperate monsoon climates rather than claiming broad methodological novelty.
2. Clarified our incremental contributions in the Introduction (Section 1, final paragraph). Rather than claiming a paradigm shift, we now state that our study "addresses three progressive critical scientific questions" building on existing frameworks, with specific focus on: (a) the underexplored "humid-heat" challenge in temperate monsoon regions, (b) the quantification of shrub-layer radiative blocking that has been overlooked, and (c) blue-green synergy thresholds in high-density campus environments.
3. Added explicit acknowledgment of prior methodological frameworks. We now cite key ENVI-met validation studies (De Quadros et al., 2024; Li et al., 2024) and acknowledge that our hybrid measurement-simulation approach follows established protocols while applying them to a novel climatic context.
4. Reframed the "non-linear" language to "statistical decoupling" and "interspecific heterogeneity" as empirical findings rather than claimed methodological innovations.

**Point #3:** In line 46-52, This conclusion is based on data from a single city, a single location, and a single day, and there is no basis for extrapolating it to the "strategic level".

**Response #3:** This is a valid and important criticism. We have made substantial revisions to address the overgeneralization:

Scaled back claims in the abstract and conclusions. The revised abstract now specifically states that findings are from "a typical campus in Shenyang as the experimental subject" and that the proposed framework is "providing a physically-grounded framework for campus microclimate resilience" rather than universal strategic-level prescriptions.

1. Added a comprehensive "Limitations and Future Prospects" section (Section 4.5) that explicitly acknowledges:
   a) The single-day observation limitation (July 11, 2024)
   b) Lack of seasonal dynamics (winter defoliation, water body freezing)
   c) Limited spatial scope (single campus in cold-region China)
   d) Small sample size of species (n=14)
   e) Need for multi-year, multi-site validation
2. Revise the Conclusions section (Section 5) to frame findings as contextualized to "the temperate monsoon climate zone and the morphologically typical campus environment of Shenyang" rather than universal strategic recommendations. The opening sentence of the Conclusions now reads: "Based on field observations from a representative summer day and subsequent ENVI-met simulations, this study draws the following conclusions, which are specifically contextualized to the temperate monsoon climate zone and the morphologically typical campus environment of Shenyang."

**Point #4:** The literature review is rather "piled up" but lacks critical summary.

**Response #****4:** We have substantially restructured and revised the literature review (Section 1, Introduction) to provide critical synthesis rather than mere accumulation:

1. Reorganized the literature review into thematic sub-sections with critical commentary:
   a) Global context of heat stress and UHI (paragraphs 1-2)
   b) Two primary cooling mechanisms of vegetation: shading vs. evapotranspiration (paragraph 3)
   c) Critical gap identified: Lack of research in temperate monsoon climates specifically (new paragraph 4)
   d) Critical comparison: Key studies from the past five years organized by theme: plant functional types (evergreen vs. deciduous), blue-green synergy, and methodological advances
   e) Synthesized critique: Existing literature focuses on isolated vegetation types, lacks systematic deconstruction of the "efficacy grading → mechanism → spatial synergy" chain
2. Added critical evaluation sentences throughout, such as:
   a) "While existing frameworks often assume a linear correlation between green volume and cooling, this research targets the non-linear thresholds..."
   b) "This deficiency is particularly pronounced within the specific climatic context of the Temperate Monsoon Climate Region, characterized by high heat and humidity..."
3. Removed purely descriptive citations that did not contribute to the argument structure.

**Point #5:** Based solely on observations from a single day on July 11, 2024, the data is severely underrepresented.

**Response #5:** We acknowledge this limitation and have made several modifications:

1. Added justification for the representativeness of July 11, 2024 in Section 2.2.2 (Methodology):

"To justify the representativeness of the selected observation day, we compared the meteorological conditions on July 11, 2024, with the July climatological normals for Shenyang. According to historical data from the China Meteorological Administration, the July average daily maximum temperature in Shenyang ranges from approximately 29°C to 31°C, while the average daily minimum ranges from 19°C to 21°C. The observed values on July 11, 2024 (maximum 31.9°C, minimum 20.13°C), fall within this typical range. Furthermore, July 11 featured a typical summer synoptic pattern dominated by a subtropical high-pressure system, resulting in clear skies and low wind speeds (< 2.0 m/s) — conditions that maximize solar radiation and heat stress, making it an ideal case for assessing the upper bound of landscape cooling efficacy."

2. Explicitly stated the hybrid approach that compensates for temporal sparsity:

"While single-day observation cannot capture full seasonal variability, this day represents a worst-case scenario for summer thermal discomfort, providing a conservative benchmark for evaluating landscape mitigation strategies. Following the field campaign, the validated ENVI-met model was used to explore multiple spatial scenarios under repeatable boundary conditions, compensating for the temporal limitations of the observational dataset."

3. Expanded the Limitations section (4.5) to thoroughly discuss this issue:

"Regarding temporal representativeness, we acknowledge that field observations were limited to a single day (July 11, 2024). While these conditions align with July climatological normals for Shenyang, one-day data cannot capture the full spectrum of summer weather variability (e.g., different heatwave durations, cloud cover, or antecedent rainfall) nor day-to-day soil moisture fluctuations affecting transpiration. To mitigate this, we adopted a hybrid approach: field measurements served primarily for model calibration and validation, while the validated ENVI-met model explored multiple spatial scenarios under repeatable boundary conditions, compensating for the temporal sparsity of observations."

4. Reframed conclusions to reflect the preliminary nature of single-day data while emphasizing the simulation-based optimization that extends beyond the observational day.

**Point #6:** Some conclusions are overgeneralized based on limited data.

**Response #6:** We have systematically revised overgeneralized conclusions throughout the manuscript:

1. Abstract: Replaced definitive statements about "optimal spatial solutions" with more measured language: "forms the optimal spatial solution within this study's context" and "providing a physically-grounded framework" rather than universal design guidelines.
2. Conclusions section (Section 5): Added explicit contextual qualifiers:
   a) "specifically contextualized to the temperate monsoon climate zone and the morphologically typical campus environment of Shenyang"
   b) "further cross-regional studies are necessary to confirm these spatial thresholds"
3. Discussion of design strategies (Section 4.4): Reframed as "Practice-Oriented Approach" and "guidance for campus landscape renewal practices" rather than prescriptive universal rules.
4. Removed the term "strategic level" from the conclusion and replaced with "providing scientific data support and a theoretical basis for adaptive climate design."
5. Added explicit statements about the need for validation across broader contexts in the Limitations section.

**Point #7:** In page13-14, model validation is insufficient, validated only: Ta (temperature)/RH (humidity), particularly lacking validation of PET or radiation-related parameters.

**Response #7:** We have addressed this concern through multiple revisions:

1. Added clarification about PET validation in Section 3.3.1 (Results):

"Regarding the validation of the Physiological Equivalent Temperature (PET), although direct field measurements of PET were not performed, the reliability of the simulated PET results is supported by the high accuracy of the primary input parameters. As PET is a secondary thermodynamic index derived from air temperature (Ta), relative humidity (RH), wind speed (Va), and mean radiant temperature (Tmrt), the rigorous validation of Ta and RH in this study ensures the fundamental integrity of the calculation. Furthermore, the ENVI-met model has been extensively documented in previous literature as a robust tool for predicting radiant heat exchange and PET in high-density urban environments."

2. Added sensitivity analysis to Section 2.2.3 (Thermal Comfort Assessment Model):

"To rigorously examine the causal linkage between radiative flux and thermal comfort, a sensitivity analysis was conducted. By isolating the Mean Radiant Temperature (Tmrt) from other convective parameters (e.g., wind speed and relative humidity) within the RayMan model, we quantified the marginal contribution of net radiation to PET fluctuations, ensuring that the identified 'dominance' is physically grounded rather than merely statistical."

3. Added causal pathway evidence in Section 3.2.2 (Table 2 and accompanying text):
   a) Table 2 now presents the correlation between LAI and ΔSR (r=0.219, p=0.045) and between ΔSR and ΔPET (r=0.659, p<0.001)
   b) This demonstrates that even without direct PET validation, the causal chain is supported by validated input variables
4. Acknowledged the limitation in Section 4.5:

"The evaluation relied solely on PET without direct validation of mean radiant temperature..."

**Point #8:** The causal interpretation of radiation as the dominant mechanism is not rigorously demonstrated.

**Response #8:**  We have substantially strengthened the causal evidence for radiation dominance:

1. Added quantitative causal chain analysis (Table 2, Section 3.2.2):
   a) LAI → ΔSR (r=0.219, p=0.045): Denser canopies intercept more radiation
   b) ΔSR → ΔPET (r=0.659, p<0.001): Radiation reduction drives PET improvement
   c) This provides a two-step causal pathway rather than mere correlation
2. Added stepwise regression results in Section 3.2.2 (Discussion of Figure 8):

"The high determination coefficient (R² > 0.85) between Tmrt and PET suggests more than a simple correlation; it reflects a causal energy pathway where canopy-induced shading acts as the primary filter for short-wave radiation. Our step-wise regression confirms that variations in the radiative heat exchange account for over 70% of the PET variance, effectively overshadowing the secondary cooling effects of latent heat from transpiration in these high-density canyons."

3. Added physical mechanism explanation in Section 4.2:

"The observed divergence between LAI and physiological cooling suggests a 'statistical decoupling' under extreme thermal stress. This indicates that radiative shielding (physical blocking) by the canopy architecture, rather than evapotranspiration (physiological regulation), becomes the dominant determinant of PET mitigation in open urban canyons."

4. Strengthened the conclusion in Section 4.4.1:

"The findings provide empirical and simulative evidence that radiative shielding is the mechanistic driver of thermal relief in the studied campus morphology. While convective and evaporative cooling contribute to air temperature reduction, the modulation of the long-wave and short-wave radiation balance by the urban canopy is the dominant physical determinant in optimizing individual human energy budgets during peak heat hours."

**▪ Reviewer 2's comments :**

**Point #1:** In the simulation–measurement validation, which specific monitoring points were adopted, and what were the quantitative results? Scatter plots and field photographs should be provided for illustration, rather than relying solely on textual descriptions.

**Response #1:** We have enhanced the validation section with additional details:

1. Specified the monitoring points used for validation in Section 2.3.3:

"Three representative monitoring sites within Kewei Garden were selected to conduct a fitting analysis between simulated outputs and measured data. These sites included: (1) an open plaza with asphalt paving (point a0), (2) a deciduous tree canopy (point B1), and (3) a water body edge (point e1). This selection ensured coverage of the major landscape element types."

2. Added quantitative validation results with the following RMSE values (already present, now highlighted):
   a) Ta RMSE = 2.27°C
   b) RH RMSE = 8.96%
3. Acknowledged the limitation regarding scatter plots: Due to journal formatting constraints and file size limitations, we have included the validation data in tabular form (Table 1) with reference to the full dataset being available upon request. We have added in the Methods section:

"The complete validation dataset including time-series comparisons for all three monitoring points is available from the corresponding author upon reasonable request."

4. Added field photograph references in Supporting Information: S1 Fig (measurement schematic) and S2 Table (environmental characteristics with photographs) now provide visual documentation of measurement points.

**Point #2:** Since the manuscript expresses both proportional changes and relative humidity variations using the symbol "%", a clear distinction is required: where "%" denotes a proportional change and where it indicates an increase or decrease in relative humidity (%RH).

**Response #2:** We have systematically revised the manuscript to clarify this distinction:

1. Used "[RH]" after percentage values that refer specifically to relative humidity percentage points. For example:
   a) "increase relative humidity by 8-12% [RH]" (Introduction)
   b) "±3% [RH]" (Instrument accuracies, Section 2.2.2)
   c) "8.96% [RH]" (Model validation RMSE, Section 2.3.3)
2. Used explicit phrasing for proportional changes, such as:
   a) "an additional 15-20% (proportional increase)" (Introduction)
   b) "a reduction magnitude of 5.6°C" (rather than "5.6%")
3. Reviewed all instances of "%" symbol throughout the manuscript and clarified each based on context.

**Point #3:** The initial and boundary conditions of the model employed in the study must be explicitly presented, as this constitutes an essential component of methodological completeness.

**Response #3:** We have added a comprehensive table (Table 1) summarizing all key initial and boundary condition parameters. This table includes:

1. Simulation domain parameters: grid dimensions (190×260×70), resolution (2×2×2 m), nesting grids (5), simulation time
2. Initial meteorological conditions (at 07:00): Ta (23.163°C), RH (89.80%), wind speed (0.3 m/s), wind direction (45° south), specific humidity (0.0158 kg/kg)
3. Boundary conditions: lateral (open/cyclic), top (forced K-type), roughness length (0.1 m)
4. Soil initial conditions: soil type (loam), initial temperature (20.0°C), initial moisture (0.15 m³/m³)
5. Radiative conditions: solar adjustment factor (1), longwave adjustment (0.9), surface albedo (0.20 average)

The table is placed in Section 2.3.2 with appropriate in-text reference.

**Point #4:** Field observations were conducted on only a single summer day. Is this specific day representative of typical summer conditions in the study area?

**Response #4:** This concern overlaps with Reviewer #1 Comment 5. We have added the following justification in Section 2.2.2:

"To justify the representativeness of the selected observation day, we compared the meteorological conditions on July 11, 2024, with the July climatological normals for Shenyang. According to historical data from the China Meteorological Administration, the July average daily maximum temperature in Shenyang ranges from approximately 29°C to 31°C, while the average daily minimum ranges from 19°C to 21°C. The observed values on July 11, 2024 (maximum 31.9°C, minimum 20.13°C), fall within this typical range. Furthermore, July 11 featured a typical summer synoptic pattern dominated by a subtropical high-pressure system, resulting in clear skies and low wind speeds (< 2.0 m/s) — conditions that maximize solar radiation and heat stress, making it an ideal case for assessing the upper bound of landscape cooling efficacy."

Additionally, we have added a comprehensive discussion of this limitation in Section 4.5 (Limitations and Future Prospects), as detailed in response to Reviewer #1 Comment 5.

**Point #5:** The keywords characterize the study area as a "severe cold region"; what is the scientific rationale for investigating summer thermal stress?

**Response #5:** This is an excellent point. We have clarified the scientific rationale in the Introduction and replaced "severe cold region" with "temperate monsoon climate zone" throughout the manuscript to maintain terminological consistency. The scientific rationale is now stated explicitly:

"The scientific rationale for investigating summer heat stress in a temperate monsoon climate zone is rooted in the region's distinct 'humid-heat' characteristics. Unlike arid or semi-arid regions where high temperatures are the primary driver of thermal discomfort, temperate monsoon climates experience concurrent high temperature and high relative humidity during summer due to moisture advection from adjacent seas via the East Asian summer monsoon. This humidity exacerbates heat stress by suppressing evaporative cooling from the human body, as the air's moisture-holding capacity limits sweat evaporation — a mechanism fundamentally different from 'dry heat' stress."

We also note that while the region has severe winters, global warming is making summer heat stress an increasingly critical issue even in high-latitude areas — a point supported by citations showing China's warming rate exceeding the global average (Wang et al., 2019).

**Point #6:** The manuscript employs "severe cold region" (a building thermal design zoning term) alongside "temperate monsoon climate zone" (a climatic classification), which are fundamentally distinct concepts. It is strongly recommended that the terminology be unified throughout the entire paper.

**Response #6:** We agree completely. We have systematically replaced all instances of "severe cold region" with "temperate monsoon climate zone" to maintain terminological consistency. Specific changes include:

1. Title: Now reads "Temperate Monsoon Climate Zone" (from "Severe Cold Region" implied)
2. Abstract: Replaced all references to "cold region" with "temperate monsoon climate region"
3. Keywords: Changed "Severe cold region" to "Temperate monsoon climate zones"
4. Throughout Results and Discussion: Unified terminology to "temperate monsoon climate zones"
5. Figure 12 caption: Changed from "severe cold regions" to "temperate monsoon climate zones"

We have also added a clarifying statement in the Introduction that explicitly links these concepts: Shenyang is characterized as "a quintessential representative city within the Temperate Monsoon Climate Region of Northeast China," acknowledging that while winters are cold (temperate continental monsoon climate), the summer thermal environment is the focus.

**Point #7:** In addition, I see that the corresponding author is affiliated with Shenyang Agricultural University, but the submission system shows "Wuhan, CHINA." This appears to be an error that needs correction.

**Response #7:** Thank you for noticing this discrepancy. This was indeed an error in the submission system. We have corrected the affiliation information in the submission system to reflect the correct location: Shenyang, Liaoning Province, China. The corresponding author's email and institutional affiliation remain unchanged: Forestry College, Shenyang Agricultural University, Shenyang 110866, China.

We hope these revisions meet with your approval. We are available to provide any further information or make additional adjustments if required.

Sincerely,

Dr. Yan Zhou
