# Supplementary material for: Microclimatic regulation effects of landscape elements on university campus in a temperate monsoon climate zone: A case study during extreme summer heat
Source: PLoS One. 2026 Nov 5;21(11):e0362099. doi: 10.1371/journal.pone.0362099 (PMC13739154; doi:10.1371/journal.pone.0362099)
Supplement: Peer review history

# Peer review: Decision Letter 0

**DOI:** 10.1371/journal.pone.0362099.r001

**Authors:** Xuan Ma

27 Apr 2026

plosone@plos.org. When you're ready to submit your revision, log on to https://www.editorialmanager.com/pone/ and select the 'Submissions Needing Revision' folder to locate your manuscript file.

- A letter that responds to each point raised by the academic editor and reviewer(s). You should upload this letter as a separate file labeled 'Response to Reviewers'.
- A marked-up copy of your manuscript that highlights changes made to the original version. You should upload this as a separate file labeled 'Revised Manuscript with Track Changes'.
- An unmarked version of your revised paper without tracked changes. You should upload this as a separate file labeled 'Manuscript'.

If you would like to make changes to your financial disclosure, please include your updated statement in your cover letter. Guidelines for resubmitting your figure files are available below the reviewer comments at the end of this letter.

If applicable, we recommend that you deposit your laboratory protocols in protocols.io to enhance the reproducibility of your results. Protocols.io assigns your protocol its own identifier (DOI) so that it can be cited independently in the future. For instructions see: https://journals.plos.org/plosone/s/submission-guidelines#loc-laboratory-protocols. Additionally, PLOS ONE offers an option for publishing peer-reviewed Lab Protocol articles, which describe protocols hosted on protocols.io. Read more information on sharing protocols at https://plos.org/protocols?utm_medium=editorial-email&utm_source=authorletters&utm_campaign=protocols.

As the corresponding author, your ORCID iD is verified in the submission system and will appear in the published article. PLOS supports the use of ORCID, and we encourage all coauthors to register for an ORCID iD and use it as well. Please encourage your coauthors to verify their ORCID iD within the submission system before final acceptance, as unverified ORCID iDs will not appear in the published article. *Only*  the individual author can complete the verification step; PLOS staff *cannot*  verify ORCID iDs on behalf of authors.

We look forward to receiving your revised manuscript.

Kind regards,

Xuan Ma

Academic Editor

PLOS One

Journal Requirements:

When submitting your revision, we need you to address these additional requirements.

1.Please ensure that your manuscript meets PLOS ONE's style requirements, including those for file naming. The PLOS ONE style templates can be found at

https://journals.plos.org/plosone/s/file?id=wjVg/PLOSOne_formatting_sample_main_body.pdf and

https://journals.plos.org/plosone/s/file?id=ba62/PLOSOne_formatting_sample_title_authors_affiliations.pdf

2. In your Methods section, please provide additional information regarding the permits you obtained for the work. Please ensure you have included the full name of the authority that approved the field site access and, if no permits were required, a brief statement explaining why.

3. Please note that PLOS One has specific guidelines on code sharing for submissions in which author-generated code underpins the findings in the manuscript. In these cases, we expect all author-generated code to be made available without restrictions upon publication of the work. Please review our guidelines at https://journals.plos.org/plosone/s/materials-and-software-sharing#loc-sharing-code and ensure that your code is shared in a way that follows best practice and facilitates reproducibility and reuse.

4. We note that Figure 1,2 and S2 in your submission contain map/satellite images which may be copyrighted. All PLOS content is published under the Creative Commons Attribution License (CC BY 4.0), which means that the manuscript, images, and Supporting Information files will be freely available online, and any third party is permitted to access, download, copy, distribute, and use these materials in any way, even commercially, with proper attribution. For these reasons, we cannot publish previously copyrighted maps or satellite images created using proprietary data, such as Google software (Google Maps, Street View, and Earth). For more information, see our copyright guidelines: http://journals.plos.org/plosone/s/licenses-and-copyright.

We require you to either (1) present written permission from the copyright holder to publish these figures specifically under the CC BY 4.0 license, or (2) remove the figures from your submission:

1. You may seek permission from the original copyright holder of Figure 1,2 and S2 to publish the content specifically under the CC BY 4.0 license.

We recommend that you contact the original copyright holder with the Content Permission Form (http://journals.plos.org/plosone/s/file?id=7c09/content-permission-form.pdf) and the following text:

“I request permission for the open-access journal PLOS ONE to publish XXX under the Creative Commons Attribution License (CCAL) CC BY 4.0 (http://creativecommons.org/licenses/by/4.0/). Please be aware that this license allows unrestricted use and distribution, even commercially, by third parties. Please reply and provide explicit written permission to publish XXX under a CC BY license and complete the attached form.”

Please upload the completed Content Permission Form or other proof of granted permissions as an "Other" file with your submission.

In the figure caption of the copyrighted figure, please include the following text: “Reprinted from [ref] under a CC BY license, with permission from [name of publisher], original copyright [original copyright year].”

2. If you are unable to obtain permission from the original copyright holder to publish these figures under the CC BY 4.0 license or if the copyright holder’s requirements are incompatible with the CC BY 4.0 license, please either i) remove the figure or ii) supply a replacement figure that complies with the CC BY 4.0 license. Please check copyright information on all replacement figures and update the figure caption with source information. If applicable, please specify in the figure caption text when a figure is similar but not identical to the original image and is therefore for illustrative purposes only.

The following resources for replacing copyrighted map figures may be helpful:

USGS National Map Viewer (public domain): http://viewer.nationalmap.gov/viewer/

The Gateway to Astronaut Photography of Earth (public domain): http://eol.jsc.nasa.gov/sseop/clickmap/

Maps at the CIA (public domain): https://www.cia.gov/library/publications/the-world-factbook/index.html and https://www.cia.gov/library/publications/cia-maps-publications/index.html

NASA Earth Observatory (public domain): http://earthobservatory.nasa.gov/

Landsat: http://landsat.visibleearth.nasa.gov/

USGS EROS (Earth Resources Observatory and Science (EROS) Center) (public domain): http://eros.usgs.gov/#

Natural Earth (public domain): http://www.naturalearthdata.com/

5. We note that Figure 3 and 12 in your submission contain copyrighted images. All PLOS content is published under the Creative Commons Attribution License (CC BY 4.0), which means that the manuscript, images, and Supporting Information files will be freely available online, and any third party is permitted to access, download, copy, distribute, and use these materials in any way, even commercially, with proper attribution. For more information, see our copyright guidelines: http://journals.plos.org/plosone/s/licenses-and-copyright.

We require you to either (1) present written permission from the copyright holder to publish these figures specifically under the CC BY 4.0 license, or (2) remove the figures from your submission:

1. You may seek permission from the original copyright holder of Figure 3 and 12 to publish the content specifically under the CC BY 4.0 license.

We recommend that you contact the original copyright holder with the Content Permission Form (http://journals.plos.org/plosone/s/file?id=7c09/content-permission-form.pdf) and the following text:

“I request permission for the open-access journal PLOS ONE to publish XXX under the Creative Commons Attribution License (CCAL) CC BY 4.0 (http://creativecommons.org/licenses/by/4.0/). Please be aware that this license allows unrestricted use and distribution, even commercially, by third parties. Please reply and provide explicit written permission to publish XXX under a CC BY license and complete the attached form.”

Please upload the completed Content Permission Form or other proof of granted permissions as an "Other" file with your submission.

In the figure caption of the copyrighted figure, please include the following text: “Reprinted from [ref] under a CC BY license, with permission from [name of publisher], original copyright [original copyright year].”

2. If you are unable to obtain permission from the original copyright holder to publish these figures under the CC BY 4.0 license or if the copyright holder’s requirements are incompatible with the CC BY 4.0 license, please either i) remove the figure or ii) supply a replacement figure that complies with the CC BY 4.0 license. Please check copyright information on all replacement figures and update the figure caption with source information. If applicable, please specify in the figure caption text when a figure is similar but not identical to the original image and is therefore for illustrative purposes only.

6. We note you have included a table to which you do not refer in the text of your manuscript. Please ensure that you refer to Table 6 in your text; if accepted, production will need this reference to link the reader to the Table.

7. If the reviewer comments include a recommendation to cite specific previously published works, please review and evaluate these publications to determine whether they are relevant and should be cited. There is no requirement to cite these works unless the editor has indicated otherwise.

[Note: HTML markup is below. Please do not edit.]

Reviewers' comments:

Reviewer's Responses to Questions

**Comments to the Author**

1. Is the manuscript technically sound, and do the data support the conclusions?

Reviewer #1: Partly

Reviewer #2: Yes

**********

2. Has the statistical analysis been performed appropriately and rigorously?

Reviewer #1: N/A

Reviewer #2: Yes

**********

3. Have the authors made all data underlying the findings in their manuscript fully available?

The PLOS Data policy

Reviewer #1: Yes

Reviewer #2: Yes

**********

4. Is the manuscript presented in an intelligible fashion and written in standard English?

Reviewer #1: Yes

Reviewer #2: Yes

**********

Reviewer #1: This manuscript addresses an important topic related to outdoor thermal comfort and landscape-based heat mitigation in temperate monsoon climates. The combination of field measurements and ENVI-met simulations provides a useful dataset.

However, several critical issues need to be addressed before the manuscript can be considered for publication:

1. In line 31, the claimed “non-linear mechanisms” are not supported by appropriate analytical methods.

2. The novelty is overstated, and the study largely follows established methodological frameworks.

3. In line 46-52, This conclusion is based on data from a single city, a single location, and a single day, and there is no basis for extrapolating it to the "strategic level".

4. The literature review is rather "piled up" but lacks critical summary.

5. Based solely on observations from a single day on July 11, 2024, the data is severely underrepresented.

6. Some conclusions are overgeneralized based on limited data.

7. In page13-14, model validation is insufficient, validated only: Ta (temperature)/RH (humidity), particularly lacking validation of PET or radiation-related parameters.

8. The causal interpretation of radiation as the dominant mechanism is not rigorously demonstrated.

Substantial revision in methodology justification, result interpretation, and claim moderation is required.

Reviewer #2: The manuscript addresses a timely and practically relevant topic—summer outdoor thermal stress in high-latitude campus environments—using a hybrid approach of field microclimate observations and ENVI-met simulations. However, several methodological, terminological, and presentational issues currently compromise the scientific rigor and clarity of the work. I recommend Major Revision before the manuscript can be considered for publication.

Below are my comments：

1. In the simulation–measurement validation, which specific monitoring points were adopted, and what were the quantitative results? Scatter plots and field photographs should be provided for illustration, rather than relying solely on textual descriptions.

2. Since the manuscript expresses both proportional changes and relative humidity variations using the symbol "%", a clear distinction is required: where "%" denotes a proportional change and where it indicates an increase or decrease in relative humidity (%RH).

3. The initial and boundary conditions of the model employed in the study must be explicitly presented, as this constitutes an essential component of methodological completeness.

4. Field observations were conducted on only a single summer day. Is this specific day representative of typical summer conditions in the study area?

5. The keywords characterize the study area as a "severe cold region"; what is the scientific rationale for investigating summer thermal stress?

6. The manuscript employs "severe cold region" (a building thermal design zoning term) alongside "temperate monsoon climate zone" (a climatic classification), which are fundamentally distinct concepts. It is strongly recommended that the terminology be unified throughout the entire paper.

In addition, I see that the corresponding author is affiliated with Shenyang Agricultural University, but the submission system shows "Wuhan, CHINA." This appears to be an error that needs correction.

**********

what does this mean?). If published, this will include your full peer review and any attached files.

If you choose “no”, your identity will remain anonymous but your review may still be made public.

**Do you want your identity to be public for this peer review?**  For information about this choice, including consent withdrawal, please see our Privacy Policy

Reviewer #1: No

Reviewer #2: **Yes:**  yujun yang

**********

[NOTE: If reviewer comments were submitted as an attachment file, they will be attached to this email and accessible via the submission site. Please log into your account, locate the manuscript record, and check for the action link "View Attachments". If this link does not appear, there are no attachment files.]

To ensure your figures meet our technical requirements, please review our figure guidelines: https://journals.plos.org/plosone/s/figures

You may also use PLOS’s free figure tool, NAAS, to help you prepare publication quality figures: https://journals.plos.org/plosone/s/figures#loc-tools-for-figure-preparation.

NAAS will assess whether your figures meet our technical requirements by comparing each figure against our figure specifications.

# Author comment: Author response to Decision Letter 1

**DOI:** 10.1371/journal.pone.0362099.r002

22 May 2026

Dear Academic Editor Dr. Xuan Ma and Reviewers,

Thank you for your valuable and constructive comments on our manuscript (PONE-D-26-04695). We have carefully addressed all the comments point by point, and the detailed responses are provided in the attached "Response to Reviewers" file.

All changes made to the original manuscript are marked in red in the "Revised Manuscript with Track Changes" file. We believe the revised manuscript has been significantly improved and is now suitable for publication in PLOS ONE.

Sincerely,

Zhou Yan

Corresponding Author

Attachment Submitted filename: Response to Reviewers.doc

# Peer review: Decision Letter 1

**DOI:** 10.1371/journal.pone.0362099.r003

**Authors:** Xuan Ma

6 Aug 2026

Dear Dr. Yan Zhou

Please submit your revised manuscript by Sep 20 2026 11:59PM. If you will need more time than this to complete your revisions, please reply to this message or contact the journal office at plosone@plos.org. When you're ready to submit your revision, log on to https://www.editorialmanager.com/pone/ and select the 'Submissions Needing Revision' folder to locate your manuscript file.

- A letter that responds to each point raised by the academic editor and reviewer(s). You should upload this letter as a separate file labeled 'Response to Reviewers'.
- A marked-up copy of your manuscript that highlights changes made to the original version. You should upload this as a separate file labeled 'Revised Manuscript with Track Changes'.
- An unmarked version of your revised paper without tracked changes. You should upload this as a separate file labeled 'Manuscript'.

If you would like to make changes to your financial disclosure, please include your updated statement in your cover letter. Guidelines for resubmitting your figure files are available below the reviewer comments at the end of this letter.

If applicable, we recommend that you deposit your laboratory protocols in protocols.io to enhance the reproducibility of your results. Protocols.io assigns your protocol its own identifier (DOI) so that it can be cited independently in the future. For instructions see: https://journals.plos.org/plosone/s/submission-guidelines#loc-laboratory-protocols. Additionally, PLOS ONE offers an option for publishing peer-reviewed Lab Protocol articles, which describe protocols hosted on protocols.io. Read more information on sharing protocols at https://plos.org/protocols?utm_medium=editorial-email&utm_source=authorletters&utm_campaign=protocols.

As the corresponding author, your ORCID iD is verified in the submission system and will appear in the published article. PLOS supports the use of ORCID, and we encourage all coauthors to register for an ORCID iD and use it as well. Please encourage your coauthors to verify their ORCID iD within the submission system before final acceptance, as unverified ORCID iDs will not appear in the published article. *Only*  the individual author can complete the verification step; PLOS staff *cannot*  verify ORCID iDs on behalf of authors.

We look forward to receiving your revised manuscript.

Kind regards,

Xuan Ma

Academic Editor

PLOS One

Journal Requirements:

If the reviewer comments include a recommendation to cite specific previously published works, please review and evaluate these publications to determine whether they are relevant and should be cited. There is no requirement to cite these works unless the editor has indicated otherwise.

Please review your reference list to ensure that it is complete and correct. If you have cited papers that have been retracted, please include the rationale for doing so in the manuscript text, or remove these references and replace them with relevant current references. Any changes to the reference list should be mentioned in the rebuttal letter that accompanies your revised manuscript. If you need to cite a retracted article, indicate the article’s retracted status in the References list and also include a citation and full reference for the retraction notice.

[Note: HTML markup is below. Please do not edit.]

Reviewers' comments:

Reviewer's Responses to Questions

**Comments to the Author**

Reviewer #1: (No Response)

Reviewer #2: All comments have been addressed

**********

2. Is the manuscript technically sound, and do the data support the conclusions?

Reviewer #1: Yes

Reviewer #2: Yes

**********

3. Has the statistical analysis been performed appropriately and rigorously?

Reviewer #1: Yes

Reviewer #2: Yes

**********

4. Have the authors made all data underlying the findings in their manuscript fully available?

The PLOS Data policy

Reviewer #1: Yes

Reviewer #2: Yes

**********

5. Is the manuscript presented in an intelligible fashion and written in standard English?

Reviewer #1: Yes

Reviewer #2: Yes

**********

Reviewer #1: The limitations of the study and the scope of its generalization should be further strengthened. The core conclusions still largely depend on a single campus environment and specific summer weather conditions. It is recommended that the authors further clarify the contextual dependence and limitations of the research results in the conclusion section.

Reviewer #2: (No Response)

**********

what does this mean?). If published, this will include your full peer review and any attached files.

If you choose “no”, your identity will remain anonymous but your review may still be made public.

**Do you want your identity to be public for this peer review?**  For information about this choice, including consent withdrawal, please see our Privacy Policy

Reviewer #1: No

Reviewer #2: No

**********

[NOTE: If reviewer comments were submitted as an attachment file, they will be attached to this email and accessible via the submission site. Please log into your account, locate the manuscript record, and check for the action link "View Attachments". If this link does not appear, there are no attachment files.]

To ensure your figures meet our technical requirements, please review our figure guidelines: https://journals.plos.org/plosone/s/figures

You may also use PLOS’s free figure tool, NAAS, to help you prepare publication quality figures: https://journals.plos.org/plosone/s/figures#loc-tools-for-figure-preparation.

NAAS will assess whether your figures meet our technical requirements by comparing each figure against our figure specifications.

# Author comment: Author response to Decision Letter 2

**DOI:** 10.1371/journal.pone.0362099.r004

18 Aug 2026

Dear editor and reviewers:

We would like to thank you for your professional review work, constructive comments, and valuable suggestions on manuscript. Your time and efforts are greatly our appreciated. We have strived to improve the manuscript accordingly as listed in details below. According to the Round 1 revision suggestions provided by editor and reviewers, all authors of this manuscript agreed to accept and adopt all the suggestions after discussion, and made the following responses to the valuable suggestions provided by reviewers point by point.

Thanks very much for taking your time to review this manuscript. We really appreciate all your comments and suggestions! Please find our itemized responses in below and my revisions:

▪ Reviewer #1’s Comment:

Point #1: The limitations of the study and the scope of its generalization should be further strengthened. The core conclusions still largely depend on a single campus environment and specific summer weather conditions. It is recommended that the authors further clarify the contextual dependence and limitations of the research results in the conclusion section.

Response #1: We agree with the reviewer and appreciate this important suggestion. We have further strengthened the description of the study limitations and narrowed the scope of generalization in the revised manuscript. First, at the end of Section 4.5, we now explicitly state that the numerical effect sizes, element rankings, and configuration thresholds are conditional on the sampled campus morphology, the examined species set, and the meteorological boundary conditions used for model calibration and scenario simulation. Second, we revised the opening paragraph of Section 5 to specify that the conclusions are based on field observations from one representative summer day at a single university campus in Shenyang and on ENVI-met simulations calibrated against those observations. We also clarify that the reported effect sizes, rankings, and spatial thresholds should not be directly generalized to other campuses, urban forms, seasons, or weather conditions without further validation. Third, we replaced the former broad final statement with a context-dependent conclusion and explicitly call for additional field observations and cross-regional model validation. These revisions do not introduce new data or alter any numerical results.

Location of revision 1: Section 4.5, “Limitations and Future Prospects,” page 51, lines 902–911 (yellow-highlighted text at lines 908–911).

“Accordingly, the numerical effect sizes, element rankings, and configuration thresholds reported here should be interpreted as conditional on the sampled campus morphology, the species set examined, and the meteorological boundary conditions used for model calibration and scenario simulation.”

Location of revision 2: Section 5, “Conclusions,” pages 51–52, lines 914–923 (yellow-highlighted paragraph).

“Based on field observations conducted on one representative summer day at a single university campus in Shenyang and on ENVI-met simulations calibrated against those observations, this study draws context-specific conclusions for the examined campus morphology and meteorological boundary conditions. The findings indicate how selected landscape elements and their combinations may regulate PET during extreme summer heat in a temperate monsoon climate; however, the reported effect sizes, rankings, and spatial thresholds should not be assumed to apply directly to other campuses, urban forms, seasons, or weather conditions. Multi-site and multi-season validation is required before broader generalization. Within these boundaries, the following key conclusions were obtained:”

Location of revision 3: Section 5, “Conclusions,” pages 53–54, lines 960–965 (yellow-highlighted paragraph).

“Within the specific campus and summer conditions examined, this study provides context-dependent evidence on the microclimatic performance of landscape elements and offers testable design hypotheses for campuses in comparable temperate monsoon settings. Their transferability to other sites, urban morphologies, seasons, and meteorological conditions should be evaluated through additional field observations and cross-regional model validation.”

▪ Reviewer #2:

Comment: All comments have been addressed.

Response: We thank Reviewer #2 for confirming that all previous comments have been addressed. No additional revision request was raised by Reviewer #2 in this round.

We sincerely appreciate the Editor’s and Reviewers’ time and constructive guidance. We hope that the revised manuscript is now suitable for publication in PLOS ONE.

Sincerely,

Dr. Yan Zhou

Attachment Submitted filename: Response to Reviewers.docx

# Peer review: Decision Letter 2

**DOI:** 10.1371/journal.pone.0362099.r005

**Authors:** Xuan Ma

20 Oct 2026

Microclimatic regulation effects of landscape elements on university campus in a temperate monsoon climate zone: a case study during extreme summer heat

PONE-D-26-04695R2

Dear Dr. Yan Zhou,

We’re pleased to inform you that your manuscript has been judged scientifically suitable for publication and will be formally accepted for publication once it meets all outstanding technical requirements.

Within one week, you’ll receive an e-mail detailing the required amendments. When these have been addressed, you’ll receive a formal acceptance letter and your manuscript will be scheduled for publication.

An invoice will be generated when your article is formally accepted. Please note, if your institution has a publishing partnership with PLOS and your article meets the relevant criteria, all or part of your publication costs will be covered. Please make sure your user information is up-to-date by logging into Editorial Manager at Editorial Manager® and clicking the ‘Update My Information' link at the top of the page. For questions related to billing, please contact billing support.

If your institution or institutions have a press office, please notify them about your upcoming paper to help maximize its impact. If they’ll be preparing press materials, please inform our press team as soon as possible -- no later than 48 hours after receiving the formal acceptance. Your manuscript will remain under strict press embargo until 2 pm Eastern Time on the date of publication. For more information, please contact onepress@plos.org.

Kind regards,

Xuan Ma

Academic Editor

PLOS One

Additional Editor Comments (optional):

Reviewers' comments:

Reviewer's Responses to Questions

**Comments to the Author**

Reviewer #1: All comments have been addressed

**********

2. Is the manuscript technically sound, and do the data support the conclusions?

Reviewer #1: (No Response)

**********

3. Has the statistical analysis been performed appropriately and rigorously?

Reviewer #1: (No Response)

**********

4. Have the authors made all data underlying the findings in their manuscript fully available?

The PLOS Data policy

Reviewer #1: (No Response)

**********

5. Is the manuscript presented in an intelligible fashion and written in standard English?

Reviewer #1: (No Response)

**********

Reviewer #1: (No Response)

**********

what does this mean?). If published, this will include your full peer review and any attached files.

If you choose “no”, your identity will remain anonymous but your review may still be made public.

**Do you want your identity to be public for this peer review?**  For information about this choice, including consent withdrawal, please see our Privacy Policy

Reviewer #1: No

**********
